# Safety and efficacy of oral icotrokinra for moderate-to-severe plaque psoriasis: a systematic review and meta-analysis of randomized controlled trials

**DOI:** 10.3389/fimmu.2026.1768292

**Published:** 2026-03-06

**Authors:** Retaj S. AlJuma, Sayed Hashim, Malak Ahmed Alshamali, Shouq Alkhatlan, Danah Jamil Hammadi, Abdullah M. Alharran

**Affiliations:** 1 College of Medicine and Medical Sciences, Arabian Gulf University, Manama, Bahrain; 2 Dermatology Department, Kuwait Institute for Medical Specializations, Kuwait City, Kuwait; 3 Department of General Surgery, Sheikh Jaber Al-Ahmad Al-Sabah Hospital, Ministry of Health, Kuwait City, Kuwait; 4 School of Medicine, Royal College of Surgeons in Ireland – Medical University of Bahrain, Alsayah, Bahrain

**Keywords:** icotrokinra, investigator’s global assessment, oral icotrokinra, plaque psoriasis, psoriasis area and severity index

## Abstract

**Background:**

Biologic therapies targeting inflammatory cytokines have transformed the management of plaque psoriasis; however, their use is limited by high cost, parenteral administration, and monitoring requirements. Icotrokinra (JNJ-77242113) is a first-in-class oral peptide that selectively inhibits the interleukin-23 (IL-23) receptor and represents a potential oral alternative. We conducted a systematic review and meta-analysis to evaluate the efficacy and safety of oral icotrokinra in moderate-to-severe plaque psoriasis.

**Methods:**

A comprehensive search of PubMed, Scopus, Web of Science, and the Cochrane Library was performed to identify randomized controlled trials (RCTs) published up to November 2025. Eligible studies enrolled adolescents or adults with moderate-to-severe plaque psoriasis treated with oral icotrokinra (200 mg once daily) versus placebo. A random-effects model was applied, and dichotomous outcomes were pooled as risk ratios (RRs) with 95% confidence intervals (CIs). Risk of bias was assessed using RoB 2, and trial sequential analysis (TSA) was performed to evaluate the conclusiveness of evidence.

**Results:**

Five RCTs comprising 1,951 participants were included. At week 16, icotrokinra significantly improved Investigator’s Global Assessment (IGA) 0/1 (RR = 7.27, 95% CI 5.62–9.40) and Psoriasis Area and Severity Index (PASI) 75 responses (RR = 6.70, 95% CI 5.20–8.62) compared with placebo (both *p* < 0.001). Higher levels of skin clearance were also achieved, including PASI 90 (RR = 13.82, 95% CI 8.75–21.84) and PASI 100 (RR = 31.65, 95% CI 12.56–79.76). Significant benefits were observed in scalp-specific disease (ss-IGA; RR = 4.27) and patient-reported outcomes, including complete symptom resolution on the Psoriasis Symptom Scale Diary (RR = 9.76). Adverse event rates did not differ significantly between icotrokinra and placebo, and heterogeneity across outcomes was minimal. TSA indicated that current evidence remains insufficient to confirm definitive conclusions.

**Conclusion:**

Oral icotrokinra demonstrates potential efficacy across multiple clinical and patient-reported endpoints with a safety profile comparable to placebo in moderate-to-severe plaque psoriasis. However, TSA indicates that the required information size has not been reached. Therefore, current evidence remains insufficient to draw definitive conclusions. Future RCTs with long-term follow-up are required to confirm these findings.

**Systematic Review Registration:**

https://www.crd.york.ac.uk/prospero/display_record.php?RecordID=1237937, identifier CRD420251237937

## 1 Introduction

Psoriasis is a chronic immune-mediated disease, affecting 125 million individuals worldwide, with 90% of cases being plaque psoriasis (1, 2). Beyond skin lesions, it impairs health-related quality of life and increases the risk of comorbidities such as psoriatic arthritis, depression, metabolic syndrome, and cardiovascular disease. The moderate-to-severe stage (one-third of patients) typically needs systemic treatment to manage skin inflammation, decrease comorbidities, and enhance overall wellbeing (3).

The enhanced understanding of pathogenesis has transformed management, and biologic agents targeting cytokines such as tumor necrosis factor-alpha (TNF-α) or interleukins (IL)-17 and IL-23 are now central to treatment. Among these, monoclonal antibodies that inhibit the p19 subunit of IL-23 (such as guselkumab and risankizumab) demonstrate high efficacy and favorable long-term safety, achieving Psoriasis Area and Severity Index (PASI) 90 or 100 responses (3). Therefore, guidelines (Joint AAD-NPF) endorse biologics as first-line systemic agents for severe disease or for psoriasis involving high-impact parts, including the scalp, genitalia, and palms/soles (4).

However, there are limits to the widespread use of biologics, including the cost, parenteral administration, and monitoring requirements (5). An additional important challenge is the parenteral route, which carries risks like needle aversion, injection-site reactions, storage constraints, and suboptimal uptake or adherence (6–8). Hence, oral systemic therapies are still needed in routine practice, particularly for individuals who prefer non-injectable options or as step-up treatment. Traditional oral agents (methotrexate, cyclosporine) and newer molecules (apremilast, a phosphodiesterase-4 inhibitor, and deucravacitinib, a tyrosine kinase 2 inhibitor) are still used (3).

Non-biologic oral treatments, however, are generally characterized by modest efficacy or safety concerns. Network meta-analyses consistently show that advanced oral agents are less effective than injectable biologics for achieving high-level skin clearance and clinically meaningful improvements in quality of life (9). Conventional oral agents (including methotrexate, cyclosporine, and acitretin) are effective for many patients but are constrained by long-term toxicities and variable tolerability (3). Consequently, there is sustained interest in novel oral agents that can balance efficacy, safety, and convenience (5).

Icotrokinra (JNJ-77242113) is a first-in-class oral peptide that selectively inhibits IL-23 binding to the IL-23 receptor, leading to downstream effector pathways of psoriatic pathogenesis (10). In early-phase clinical trials, icotrokinra showed dose-dependent improvements in skin clearance, durable responses, and a safety profile comparable to placebo through at least 1 year of follow-up. Recent phase 3 randomized controlled trials (RCTs), including ICONIC-LEAD, ICONIC-ADVANCE 1/2, and ICONIC-TOTAL (11–13), have consistently reported the efficacy and safety of icotrokinra in adults and adolescents with moderate-to-severe plaque psoriasis and in those with high-impact site involvement.

Despite these emerging data, the overall efficacy and safety of icotrokinra remain uncertain. To date, no comprehensive synthesis has integrated efficacy and safety outcomes across RCTs or quantitatively assessed the magnitude and consistency of treatment effects. Therefore, this systematic review and meta-analysis aimed to evaluate the efficacy and safety of oral icotrokinra for moderate-to-severe plaque psoriasis and to determine the magnitude and consistency of treatment effects across RCTs.

## 2 Methods

### 2.1 Study registration

The study protocol was registered on PROSPERO (CRD420251237937). This systematic review adhered to the Preferred Reporting Items for Systematic Reviews and Meta-Analyses (PRISMA) statement standards (14) and is in accordance with the guidelines of the Cochrane Handbook for Systematic Reviews (15). The PRISMA checklist is provided in **Supplementary File 1**.

### 2.2 Literature search and study selection

In November 2025, we carried out a thorough search of the Web of Science, Cochrane Library, PubMed, and Scopus databases using the terms (Icotrokinra OR “IL-23 receptor antagonist” OR JNJ-2113 OR JNJ-77242113) AND (“Plaque Psoriasis” OR “Psoriasis Vulgaris”). Detailed search strategies are displayed in **Supplementary Table 1**.

Two different authors independently reviewed the search results to determine which papers met the inclusion and exclusion criteria. They evaluated the qualifying conditions for each study as well. Following our initial screening of abstracts and titles, we got full texts of possibly relevant publications for further assessment. We revised the references to find potential papers that fulfilled our inclusion criteria. Any conflicts that arose throughout the screening process were resolved via consensus.

### 2.3 Eligibility criteria

We included studies that met the following PICO criteria:

- Population: adolescents (12–18 years) and adults (≥18 years) with moderate-to-severe plaque psoriasis.
- Intervention: oral icotrokinra at a daily dose of 200 mg.
- Control: placebo.
- At least one of the following outcomes: Investigator’s Global Assessment (IGA) 0/1 response, IGA 0 response, Psoriasis Area Severity Index (PASI) 75 response, PASI 90 response, PASI 100 response, area-specific improvements, Psoriasis Symptom Scale Diary (PSSD) symptom scores, or adverse events.
- Study design: RCTs.

We excluded studies that did not meet any part of our predetermined inclusion criteria.

### 2.4 Data extraction

Data from the selected research studies were retrieved by two authors using an Excel spreadsheet. We extracted data on the study’s design, country, sample size, population, inclusion criteria, intervention, control, and follow-up time. We also gathered demographic information (age, gender, and race), clinical data [body mass index (BMI), psoriasis duration, PASI scores, IGA severity, body surface area (BSA) involvement], and past treatment history. The extracted primary outcomes included IGA responses, PASI responses, area-specific improvement, PSSD symptom scores, and adverse events. The study of Gold et al. included data from two trials (ICONIC-ADVANCE 1 and 2) (12). We did not analyze data from FRONTIER-2 (16), as it was a long-term follow-up trial of FRONTIER-1 (17).

### 2.5 Quality assessment and certainty of evidence

Two authors used the Cochrane tool for RCTs (RoB 2) (18) to evaluate the quality of the included studies. The Robvis web tool was used to create quality assessment figures (19). Discussion was used to resolve any disputes. We used the Grading of Recommendations, Assessment, Development, and Evaluation (GRADE) tool to assess the quality of evidence in reported outcomes (20).

### 2.6 Outcome definition

For IGA responses, 0 indicated clear skin, and 1 indicated almost clear skin. For PASI 75, 90, and 100 responses, 75/90/100 indicated percentage improvement in disease severity. A clinically meaningful improvement in PSSD itch score was defined as a score improvement of ≥4 points from baseline.

### 2.7 Statistical analysis

We used R 4.5.0 (21) with RStudio 2024.12.1 + 563 (22). Meta-analyses were conducted using the meta and metafor R packages. Dichotomous data were analyzed using risk ratios (RRs) and 95% confidence intervals. Dichotomous outcomes were pooled using the inverse-variance weighting method, and between-study heterogeneity was estimated using the DerSimonian–Laird random-effects model. Visual inspection of the forest plot was used to measure statistical heterogeneity between trials, in addition to *I*-squared (*I*^2^) and chi-squared (*χ*2) statistics. *I*^2^ values of 50% indicated significant heterogeneity. All results were analyzed using a random-effects model. We utilized the Egger test (23) and funnel plot to assess publication bias and small-study effects where there were 10 or more studies; otherwise, we used the Doi plot and the Luis Furuya-Kanamori (LFK) index (24). In case of major asymmetry and significant small-study effect, we also used the trim-and-fill method to estimate the number of missing studies and update the pooled estimates (25).

Trial Sequential Analysis (TSA) was performed to control for the risks of random errors due to sparse data and repeated significance testing in cumulative meta-analysis. The analysis was conducted using Trial Sequential Analysis Viewer (version 0.9.5.10 Beta; Copenhagen Trial Unit, Centre for Clinical Intervention Research, Rigshospitalet, Copenhagen, Denmark) (26). A two-sided design was applied with a type I error (*α*) of 5% and a type II error (*β*) of 20% (power = 80%). Sequential monitoring boundaries for benefit and harm were constructed using the O’Brien–Fleming alpha-spending function with the information axis based on the cumulative sample size. For dichotomous outcomes, the required information size (RIS) was calculated using a random-effects model with a variance-based heterogeneity correction. The control event proportion was derived from the pooled control groups of the included trials for each outcome. The anticipated intervention effect was defined according to the relative risk reduction estimated from the cumulative meta-analysis. The RIS was therefore automatically adjusted for between-study heterogeneity by the software. The cumulative *Z*-curve was plotted against the trial sequential monitoring boundaries and the RIS to determine whether the available evidence was sufficient to confirm or reject a predefined intervention effect or whether additional trials were required. The ordering of trials in the cumulative analysis followed their chronological inclusion in the dataset. Futility boundaries were not applied.

## 3 Results

### 3.1 Literature search

**Figure 1:**
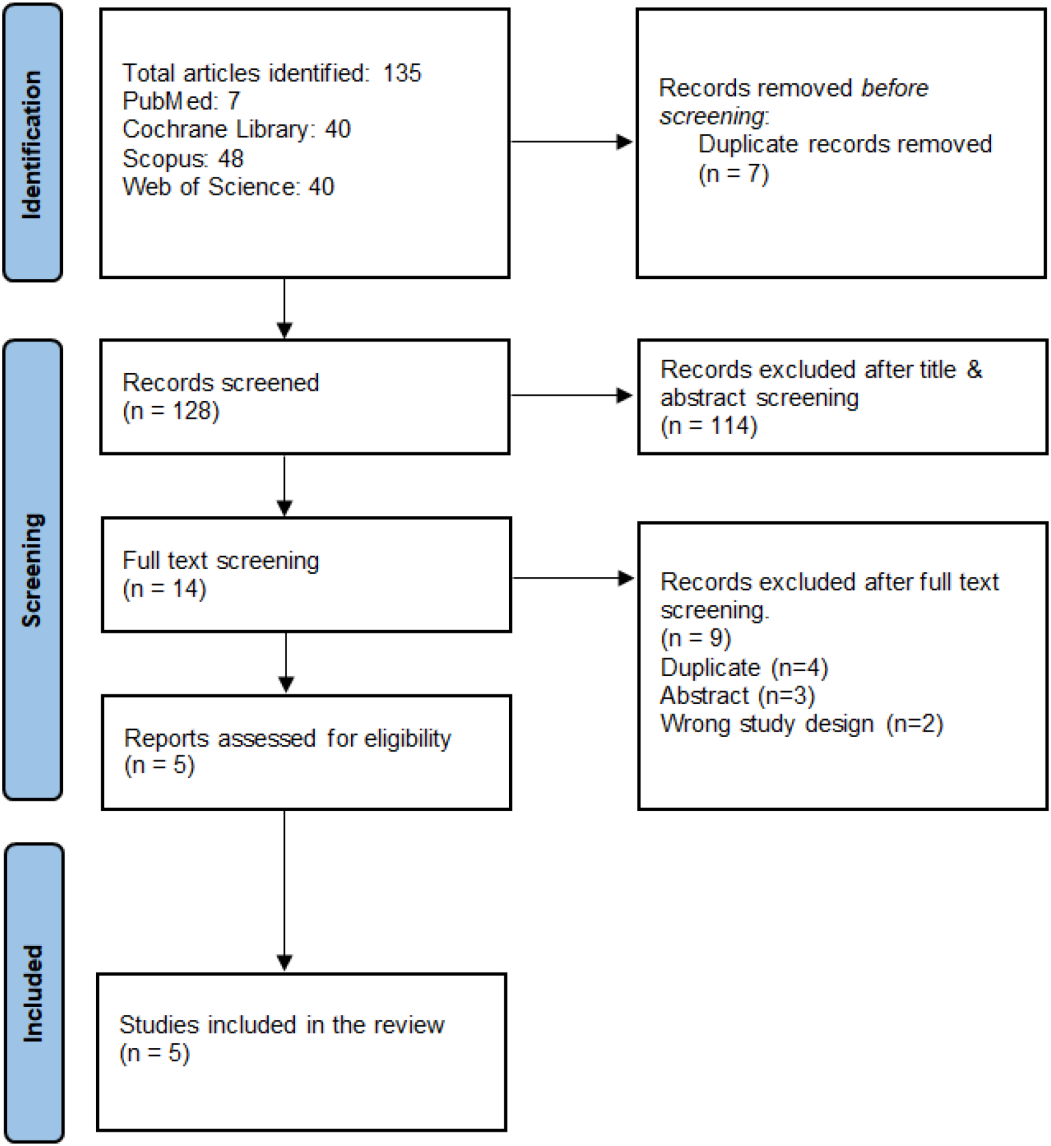
PRISMA flowchart for the systematic search and selection process.

We conducted a thorough literature search using PubMed, Scopus, Web of Science, and the Cochrane Library and found 135 articles, 7 of which were duplicates. The remaining 128 articles were assessed using the title and abstract, and 114 were excluded because they did not match the inclusion criteria. Nine of the 14 articles that underwent full-text screening were excluded, leaving only five RCTs (11–13, 16, 17) for inclusion (**Figure 1**).

### 3.2 Study characteristics

We included five RCTs from five published original studies: the FRONTIER-1 and -2 (the same trial), ICONIC-LEAD, ICONIC-ADVANCE 1 and 2, and ICONIC-TOTAL studies (11–13, 16, 17). The study comprised 1,951 adult patients with moderate-to-severe plaque psoriasis, consisting of 1,339 in the icotrokinra group and 612 in the placebo group. The mean age of the pooled study population ranged from 42.0 to 48.4 years, with the majority of the respondents being male (58%–72%) and white (71%–86%). The patients had a lengthy history of psoriasis, with a mean disease duration of 15.2 to 21.21 years, and 70%–79% had previously received systemic therapy. The detailed characteristics of the included studies are summarized in **Table 1**, while the baseline demographic and clinical characteristics of participants are presented in **Table 2**.

**Table 1:** Study characteristics of the included studies.

Study ID	Study design	Country	Total sample size, no.	Population	Inclusion criteria	Intervention	Control	Follow-up period, weeks	Primary outcomes
Bissonnette et al., 2024 (FRONTIER-1), Ferris et al., 2025 (FRONTIER-2) (17)	Phase 2, multicenter, double-blind, placebo-controlled trial	North America, Europe, and Asia	85	Eligible adults (≥18 years of age) with moderate-to-severe plaque psoriasis (IGA score ≥3, BSA ≥10%, PASI score ≥12), diagnosed at least 6 months prior, and were candidates for phototherapy or systemic treatment	Adults ≥18 years of age, moderate-to-severe plaque psoriasis (IGA ≥ 3, BSA ≥ 10%, PASI ≥ 12), diagnosis of plaque psoriasis at least 6 months before the first dose, candidate for phototherapy or systemic psoriasis treatment	JNJ-77242113 (icotrokinra) (25 mg once daily, 25 mg twice daily, 50 mg once daily, 100 mg once daily, or 100 mg twice daily)	Placebo	16 weeks (FRONTIER-1), 52 weeks (FRONTIER-2)	PASI 75 response at week 16
Bissonnette et al., 2025 (ICONIC-LEAD) (13)	Phase 3, multicenter, double-blind, placebo-controlled trial	Argentina, Australia, Canada, China, Germany, Hungary, Italy, Japan, Poland, South Korea, Spain, Taiwan, Turkey, United Kingdom, and United States	684	Eligible adults (≥18 years of age) and adolescents (12 to <18 years of age) with moderate-to-severe plaque psoriasis (BSA ≥10%, PASI score ≥12, IGA score ≥3), diagnosed at least 26 weeks before screening, and were candidates for phototherapy or other systemic treatment	Adults ≥18 years and adolescents 12 to <18 years, moderate-to-severe plaque psoriasis (BSA ≥ 10%, PASI ≥ 12, IGA ≥ 3), diagnosis of plaque psoriasis at least 26 weeks before screening, candidate for phototherapy or other systemic psoriasis treatment	Once-daily icotrokinra (200 mg)	Placebo	16–24 weeks	IGA 0/1 response, PASI 90 response at week 16
Gold et al., 2025 (ICONIC-ADVANCE 1) (12)	Phase 3, multicenter, double-blind, placebo-controlled trial	(ICONIC-ADVANCE 1): Argentina, Australia, Brazil, Canada, Germany, Hungary, Japan, Republic of Korea, Poland, Spain, Taiwan, UK, and USA (ICONIC-ADVANCE 2): Australia, Brazil, Canada, Germany, Hungary, Japan, Republic of Korea, Poland, Romania, Spain, Taiwan, and United States	(ICONIC-ADVANCE 1): 467, (ICONIC-ADVANCE 2): 404	Eligible adults (aged ≥18 years) with moderate-to-severe plaque psoriasis diagnosed for at least 26 weeks at screening (total BSA of psoriasis involvement ≥10%, PASI score ≥12, and IGA score ≥3) and who were candidates for phototherapy or systemic treatment	Adults ≥18 years with moderate-to-severe plaque psoriasis diagnosed for at least 26 weeks at screening [total body surface area (BSA) of psoriasis involvement ≥ 10%; Psoriasis Area and Severity Index (PASI) score ≥ 12; and Investigator’s Global Assessment (IGA) score ≥ 3] and who were candidates for phototherapy or systemic treatment	Once-daily oral icotrokinra (200 mg)	Placebo, deucravacitinib (6 mg)	16 weeks	IGA 0/1 response, PASI 90 response at week 16
Gooderham et al., 2025 (ICONIC-TOTAL) (11)	Phase 3, multicenter, double-blind, placebo-controlled trial	Argentina, Canada, Germany, Hungary, Poland, South Korea, Spain, Taiwan, United Kingdom, United States	311	Adults (≥18 years of age) and adolescents (≥12 to <18 years of age) with plaque psoriasis for 26 weeks or more, who were candidates for phototherapy or systemic treatment, had an inadequate response to topical therapies, and had at least moderate psoriasis involving one or more high-impact sites (scalp, genital, and/or hand/foot)	Adults ≥18 years and adolescents ≥12 to <18 years, plaque psoriasis for at least 26 weeks, candidate for phototherapy or systemic treatment, inadequate response to one or more topical therapies, total affected BSA ≥1%, IGA score ≥2, at least moderate psoriasis in one or more high-impact sites, defined as: ss-IGA score ≥3, sPGA-G score ≥3, and/or hf-PGA score ≥3	Once-daily oral icotrokinra (200 mg)	Placebo	16 weeks	IGA 0/1 response

IGA, Investigator’s Global Assessment; BSA, body surface area, PASI; Psoriasis Area and Severity Index, ss-IGA; scalp-specific Investigator’s Global Assessment; sPGA-G, static Physician’s Global Assessment of Genitalia; hf-PGA, Physician’s Global Assessment of hands and feet.

**Table 2:** Baseline of the included studies.

Study ID	Study group	Sample size, no.	Age, mean(SD)	Male sex, no. (%)	Race (Asian), no. (%)	Race (Black), no. (%)	Race (White), no. (%)	BMI, mean(SD)	Duration of psoriasis (yr), mean (SD)	PASI total score, mean(SD)	Overall IGA score, moderate plaque psoriasis, no. (%)	Overall IGA score, severe plaque psoriasis, no. (%)	PSSD symptom score, no. (%)	Percentage affected BSA, mean (SD)	ss-IGA score ≥3, no. (%)	Previous use of any systemic therapy for psoriasis, no. (%)	Conventional nonbiologic systemic therapy, no. (%)	Biologic therapy, no. (%)	Phototherapy, no. (%)	Nonconventional nonbiologic systemic therapy, no. (%)
Bissonnette et al., 2024, (FRONTIER 1), Ferris et al., 2025, (FRONTIER 2) (17)	Icotrokinra	42	42.0 (11.34)	30 (61)	9 (21)	2 (5)	30 (71)	30.0 (5.40)	16.7 (13.78)	20.33 (6.51)	30 (71)	12 (29)	NA	24.2 (12.55)	NA	31 (74)	20 (48)	9 (21)	14 (33)	3 (7)
	Placebo	43	43.9 (14.70)	25 (58)	5 (12)	0	37 (86)	31.2 (7.61)	17.9 (14.37)	18.99 (5.34)	38 (88)	5 (12)	NA	26.1 (15.72)	NA	34 (79)	17 (40)	7 (16)	19 (44)	4 (9)
Bissonnette et al., 2025, (ICONIC-LEAD) (13)	Icotrokinra	456	42.4 (16.3)	291 (64)	110 (24)	6 (1)	329 (72)	29.2 (6.9)	17.3 (13.9)	19.4 (7.1)	341 (75)	115 (25)	50.1 (25.8)	24.6 (14.3)	344 (76)	327 (72)	217 (48)	148 (32)	136 (30)	37 (8)
	Placebo	228	43.2 (16.6)	156 (68)	57 (25)	2 (<1)	165 (72)	29.3 (7)	16.6 (12.7)	20.8 (8.1)	173 (76)	55 (24)	49.4 (25.4)	27.1 (16.2)	165 (72)	163 (71)	109 (48)	85 (37)	67 (29)	17 (7)
Gold et al., 2025, (ICONIC-ADVANCE 1) (12)	Icotrokinra	311	47.1 (13.19)	223 (72)	69 (22)	4 (1)	231 (74)	29.2 (6.31)	17.52 (11.10)	18.60 (5.33)	251 (81)	60 (19)	NA	20 (11.85)	216 (69)	236 (76)	171 (55)	86 (28)	112 (36)	22 (7)
	Placebo	156	46.9 (12.78)	105 (67)	34 (22)	3 (2)	118 (76)	29.6 (8.08)	17.88 (12.75)	17.15 (5.37)	123 (79)	33 (21)	NA	20 (12.41)	104 (46)	110 (71)	79 (51)	42 (27)	53 (34)	12 (8)
Gold et al., 2025, (ICONIC-ADVANCE 2) (12)	Icotrokinra	322	45.9 (13.78)	218 (68)	34 (11)	9 (3)	274 (85)	29.9 (6.36)	17.43 (13.38)	18 (5.26)	252 (78)	70 (22)	NA	21 (12.59)	208 (65)	225 (70)	165 (51)	78 (24)	98 (30)	16 (5)
	Placebo	82	48.4 (13.90)	55 (67)	15 (18)	2 (2)	65 (79)	29.5 (5.78)	21.21 (15.17)	17.95 (6.89)	67 (82)	15 (18)	NA	22 (13.33)	54 (66)	58 (71)	39 (48)	26 (32)	31 (38)	3 (4)
Gooderham et al., 2025, (ICONIC-TOTAL) (11)	Icotrokinra	208	45.3 (14.6)	137 (66)	41 (20)	2 (1)	161 (77)	29.0 (7) (n=203)	16.8 (13)	NA	153 (74)	46 (22)	53.2 (26.3) (n=191)	16.6 (13.5)	167 (80)	151 (73)	103 (49)	71 (34)	89 (43)	15 (7)
	Placebo	103	43.5 (13.8)	63 (61)	20 (19)	0	82 (80)	29.4 (8) (n=101)	15.2 (10.5)	NA	73 (71)	22 (21)	54.6 (26.4) (n=87)	14.8 (11.7)	85 (82)	75 (73)	55 (53)	32 (31)	32 (31)	7 (7)

BMI, body mass index; BSA, body surface area; IGA, Investigator’s Global Assessment; PASI, Psoriasis Area and Severity Index; PSSD, Psoriasis Symptoms and Signs Diary; SD, standard deviation; ss-IGA, scalp-specific Investigator’s Global Assessment; NA, not available.

### 3.3 Quality assessment and certainty of evidence

**Figure 2:**
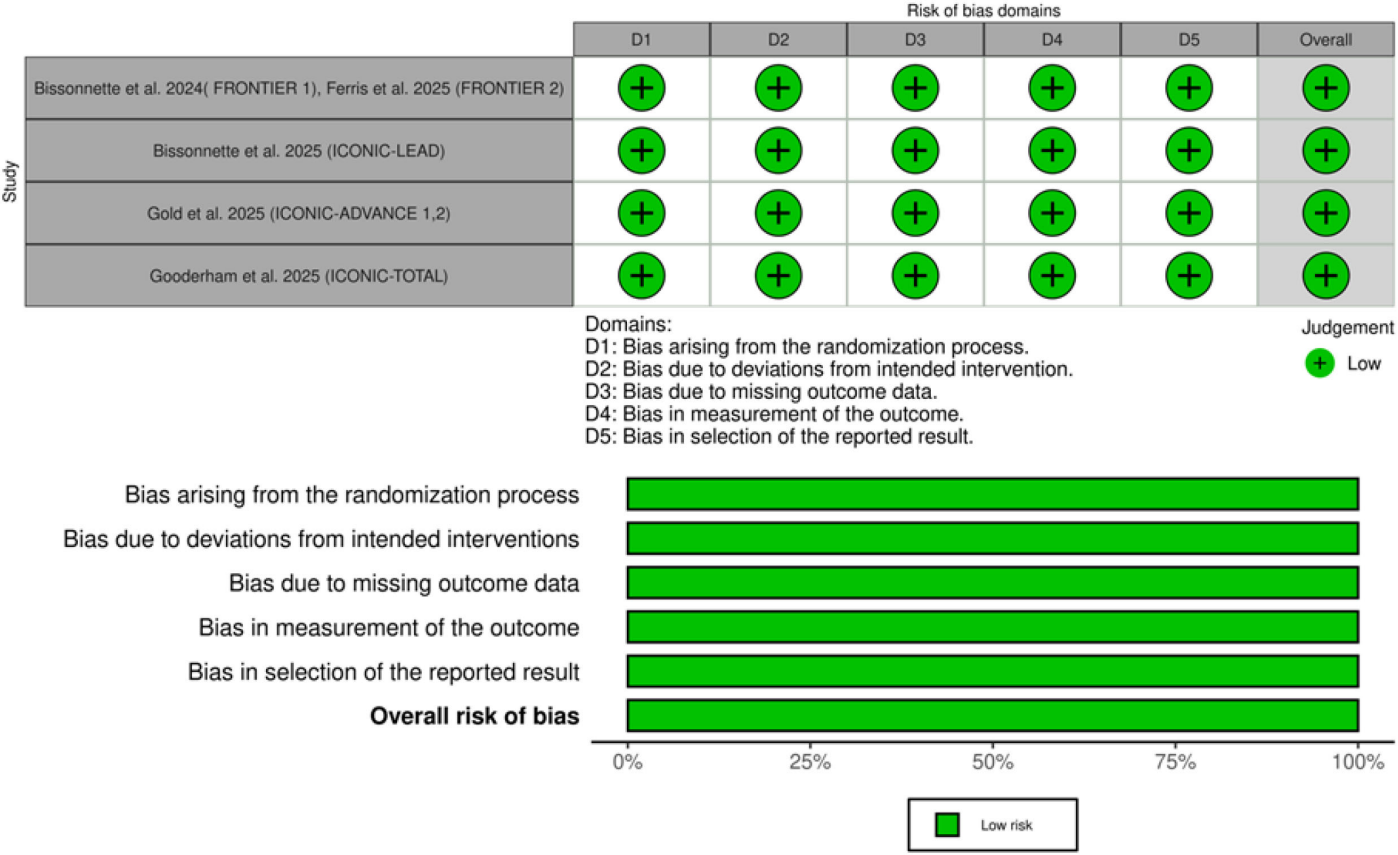
Summary and traffic light plots of the risk of bias of the included randomized controlled trials.

The included studies yielded low risk of bias in all domains, which indicated outstanding methodology with low overall risk of bias (11–13, 16, 17). **Figure 2** shows the traffic-light plot of the quality assessment. Using GRADE assessment, the certainty of evidence was moderate to high for all assessed outcomes (**Table 3**).

**Table 3:** Grading of recommendations, assessment, development, and evaluation (GRADE) evidence profile.

Certainty assessment							Study event rates (%)		Effect		Certainty
Number of studies	Study design	RoB	Inconsistency	Indirectness	Imprecision	Others	Icotrokinra	Placebo	Relative (95% CI)	Absolute (95% CI)	
IGA 0 at week 16											
5	RCTs	Not serious	Not serious	Not serious	Not serious	Publication bias strongly suspected	456/1,339 (34.1%)	8/612 (1.3%)	RR 24.11 (12.31 to 47.20)	299 more per 1,000 (from 147 more to 600 more)	⊕⊕⊕◯ Moderate
IGA 0/1 at week 16											
5	RCTs	Not serious	Not serious	Not serious	Not serious	None	880/1,339 (65.7%)	54/612 (8.8%)	RR 7.27 (5.62 to 9.40)	552 more per 1,000 (from 406 more to 739 more)	⊕⊕⊕⊕ High
PASI 75 at week 16											
4	RCTs	Not serious	Not serious	Not serious	Not serious	Publication bias strongly suspected	828/1,131 (73.2%)	55/509 (10.8%)	RR 6.70 (5.20 to 8.62)	616 more per 1,000 (from 454 more to 823 more)	⊕⊕⊕◯ Moderate
PASI 90 at week 16											
4	RCTs	Not serious	Not serious	Not serious	Not serious	Publication bias strongly suspected	606/1,131 (53.6%)	18/509 (3.5%)	RR 13.82 (8.75 to 21.84)	450 more per 1,000 (from 271 more to 730 more)	⊕⊕⊕◯ Moderate
PASI 100 at week 16											
4	RCTs	Not serious	Not serious	Not serious	Not serious	Publication bias strongly suspected	339/1,131 (30.0%)	4/509 (0.8%)	RR 31.65 (12.56 to 79.76)	245 more per 1,000 (from 92 more to 630 more)	⊕⊕⊕◯ Moderate
ss-IGA 0/1 at week 16											
4	RCTs	Not serious	Not serious	Not serious	Not serious	Publication bias strongly suspected	792/1,112 (71.2%)	80/490 (16.3%)	RR 4.27 (3.40 to 5.36)	533 more per 1,000 (from 391 more to 710 more)	⊕⊕⊕◯ Moderate
PSSD symptom score of 0 at week 16											
5	RCTs	Not serious	Not serious	Not serious	Not serious	Publication bias strongly suspected	256/1,225 (20.9%)	9/551 (1.6%)	RR 9.76 (5.06 to 18.86)	140 more per 1,000 (from 65 more to 285 more)	⊕⊕⊕◯ Moderate
PSSD itch score at week 16											
4	RCTs	Not serious	Not serious	Not serious	Not serious	None	613/1,027 (59.7%)	61/426 (14.3%)	RR 4.14 (3.27 to 5.26)	449 more per 1,000 (from 324 more to 609 more)	⊕⊕⊕⊕ High

*Downgrading of certainty of evidence in the majority of outcomes was due to suspected publication bias.

### 3.4 Safety and efficacy outcomes

#### 3.4.1 Primary efficacy outcomes

**Figure 3:**
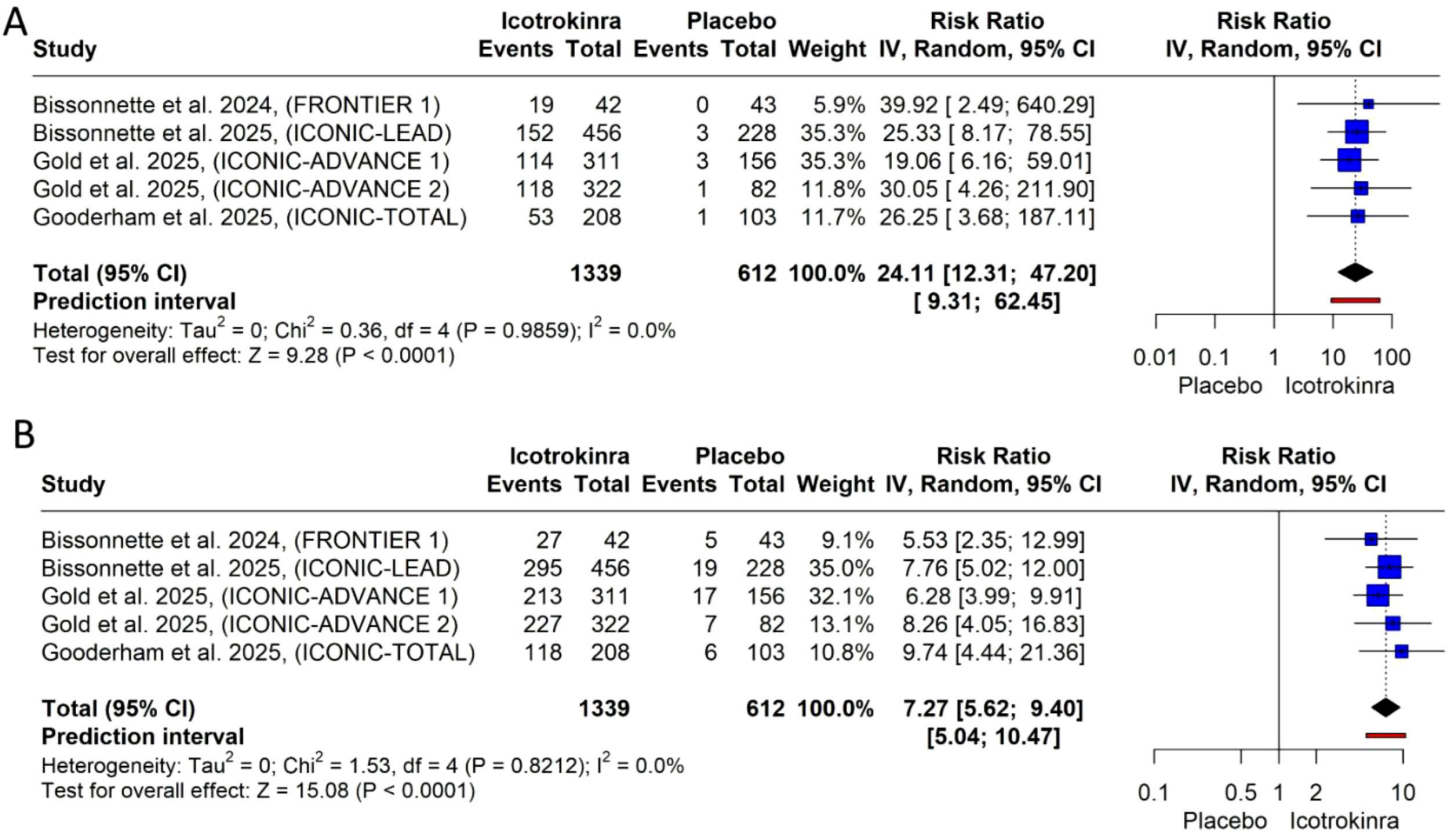
Forest plots for IGA response at week 16: **(A)** for IGA 0 and **(B)** for IGA 0/1.

IGA response at week 16: The pooled analysis demonstrated that icotrokinra significantly increased the IGA response [IGA 0 (five trials) and IGA 0/1 (four trials)] at week 16 compared to placebo [RR = 24.11, 95% CI (12.31; 47.20), *p* < 0.001; RR = 7.27, 95% CI (5.62; 9.40), *p* < 0.001, respectively) (**Figures 3A, B**). The pooled analysis was homogeneous and *I*^2^ = 0 in both IGA 0 and IGA 0/1. In IGA 0/1, the LFK index was −0.57 (no asymmetry) (**Supplementary Figure 1**); however, in IGA 0, the LFK index was 2.18 (major asymmetry) (**Supplementary Figure 2**). In IGA 0, the trim-and-fill analysis (L-estimator) was performed. Two studies were included to achieve funnel plot symmetry. The adjusted pooled relative risk remained significant (RR = 22.53, 95% CI 12.12–41.85), suggesting that the observed treatment effect is robust and unlikely to be materially influenced by publication bias (**Supplementary Table 2**; **Supplementary Figure 3**).

**Figure 4:**
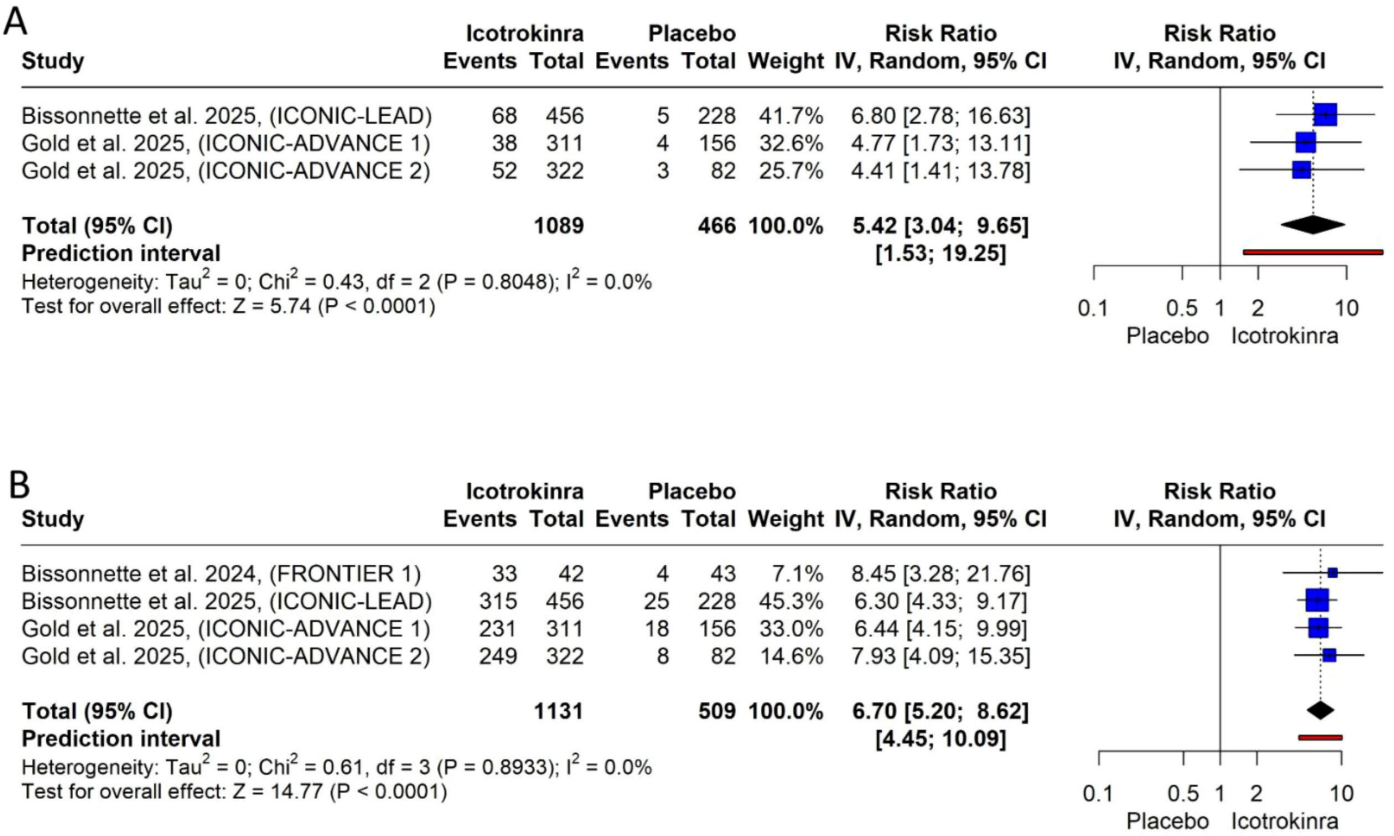
Forest plots for PASI response: **(A)** for PASI 75 response at week 4 and **(B)** for PASI 75 response at week 16.

PASI 75 response at weeks 4 and 16: The pooled analysis demonstrated that icotrokinra significantly increased the PASI 75 response at weeks 4 (three trials) and 16 (four trials) compared to placebo [RR = 5.42, 95% CI (3.04; 9.65), *p* < 0.001; RR = 6.70, 95% CI (5.20; 8.62), *p* < 0.001; respectively] (**Figures 4A, B**). The pooled analysis was homogeneous and *I*^2^ = 0 at weeks 4 and 16. At week 4, the LFK index was 0.60 (no asymmetry) (**Supplementary Figure 4**); however, at week 16, the LFK index was 4 (major asymmetry) (**Supplementary Figure 5**). At week 16, the trim-and-fill analysis (L-estimator) identified one potentially missing study. After adjustment, the pooled relative risk for PASI 75 response at week 16 remained significant (RR = 6.58, 95% CI 5.16–8.40, *p* < 0.0001), indicating that the strong treatment effect is robust and unlikely to be materially influenced by publication bias (**Supplementary Table 3**; **Supplementary Figure 6**).

**Figure 5:**
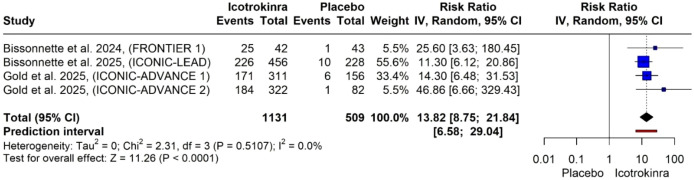
Forest plot for PASI 90 response at week 16.

PASI 90 response at week 16: The pooled analysis of four trials demonstrated that icotrokinra significantly increased the PASI 90 response at week 16 compared to placebo [RR = 13.82, 95% CI (8.75; 21.84), *p* < 0.001] (**Figure 5**). The pooled analysis was homogeneous (*I*^2^ = 0). The LFK index was 4.79 (major asymmetry) (**Supplementary Figure 7**). The trim-and-fill analysis (L-estimator) identified two potentially missing studies. After adjustment, the pooled relative risk for PASI 90 response at week 16 remained significant (RR = 12.34, 95% CI 7.99–19.05, *p* < 0.0001), indicating that the strong treatment effect is robust and unlikely to be materially influenced by publication bias (**Supplementary Table 4**; **Supplementary Figure 8**).

**Figure 6:**
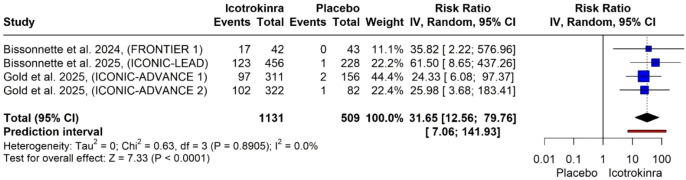
Forest plot for PASI 100 response at week 16.

PASI 100 response at week 16: The pooled analysis of four trials demonstrated that icotrokinra significantly increased the PASI 100 response at week 16 compared to placebo [RR = 31.65, 95% CI (12.56; 79.76), *p* < 0.001] (**Figure 6**). The pooled analysis was homogeneous (*I*^2^ = 0). The LFK index was 2.23 (major asymmetry) (**Supplementary Figure 9**). The trim-and-fill analysis detected no evidence of publication bias, and the pooled estimate remained highly significant (**Supplementary Table 5**; **Supplementary Figure 10**).

#### 3.4.2 Secondary efficacy outcomes

**Figure 7:**
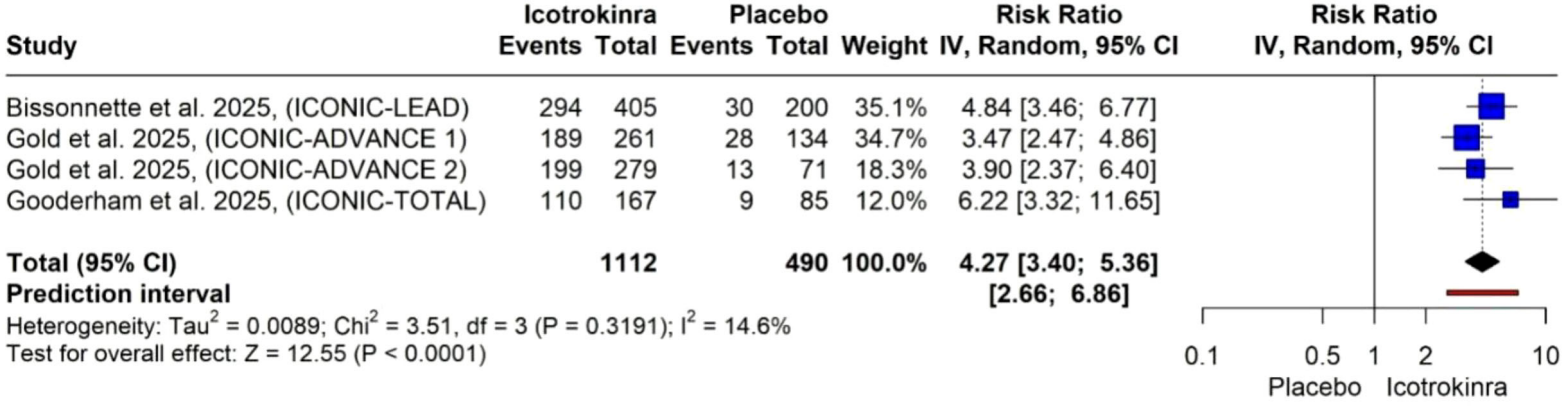
Forest plot for area-specific improvement (ss-IGA).

ss-IGA at week 16: The pooled analysis of four trials demonstrated that icotrokinra significantly increased the ss-IGA compared to placebo [RR = 4.27, 95% CI (3.40; 5.36), *p* < 0.001] (**Figure 7**). The pooled analysis was homogeneous (*I*^2^ = 0). The LFK index was 2.24 (major asymmetry) (**Supplementary Figure 11**). The trim-and-fill analysis (L-estimator) identified one potentially missing study. After adjustment, the pooled relative risk for ss-IGA remained significant (RR = 4.05, 95% CI 3.24–5.06, *p* < 0.0001), indicating that the strong treatment effect is robust and unlikely to be materially influenced by publication bias (**Supplementary Table 6**; **Supplementary Figure 12**).

**Figure 8:**
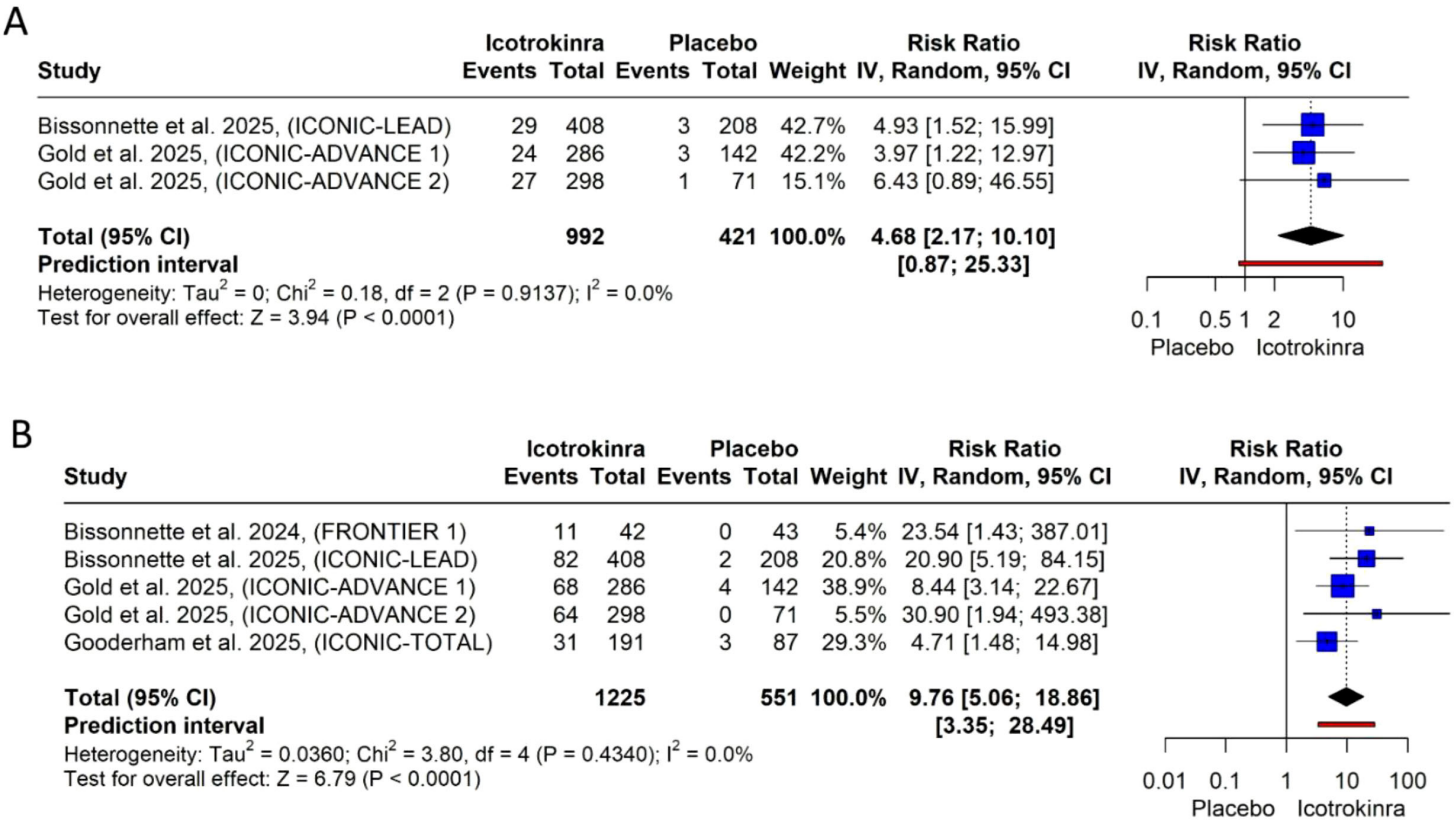
Forest plots for PSSD symptom score of 0: **(A)** for week 8 and **(B)** for week 16.

PSSD symptom score of 0 at weeks 8 and 16: The pooled analysis demonstrated that icotrokinra significantly increased the PSSD symptom score of 0 at weeks 8 (three trials) and 16 (five trials) compared to placebo [RR = 4.68, 95% CI (2.17; 10.10), *p* < 0.001; RR = 9.76, 95% CI (5.06; 18.86), *p* < 0.001, respectively) (**Figures 8A, B**). The pooled analysis was homogeneous, and *I*^2^ = 0 at weeks 8 and 16. At week 8, the LFK index was 1.01 (minor asymmetry) (**Supplementary Figure 13**); however, at week 16, the LFK index was 3.68 (major asymmetry) (**Supplementary Figure 14**). At week 8, the trim-and-fill analysis (L-estimator) identified one potentially missing study. After adjustment, the pooled relative risk for PSSD symptom score of 0 remained significant (RR = 4.42, 95% CI 2.16–9.06, *p* < 0.001), indicating that the strong treatment effect is robust and unlikely to be materially influenced by publication bias (**Supplementary Table 7**; **Supplementary Figure 15**). At week 16, the trim-and-fill analysis (L-estimator) identified two potentially missing studies. After adjustment, the pooled relative risk for PSSD symptom score of 0 remained significant (RR = 8.55, 95% CI 4.7–15.55, *p* < 0.001), indicating that the strong treatment effect is robust and unlikely to be materially influenced by publication bias (**Supplementary Table 8**; **Supplementary Figure 16**).

**Figure 9:**
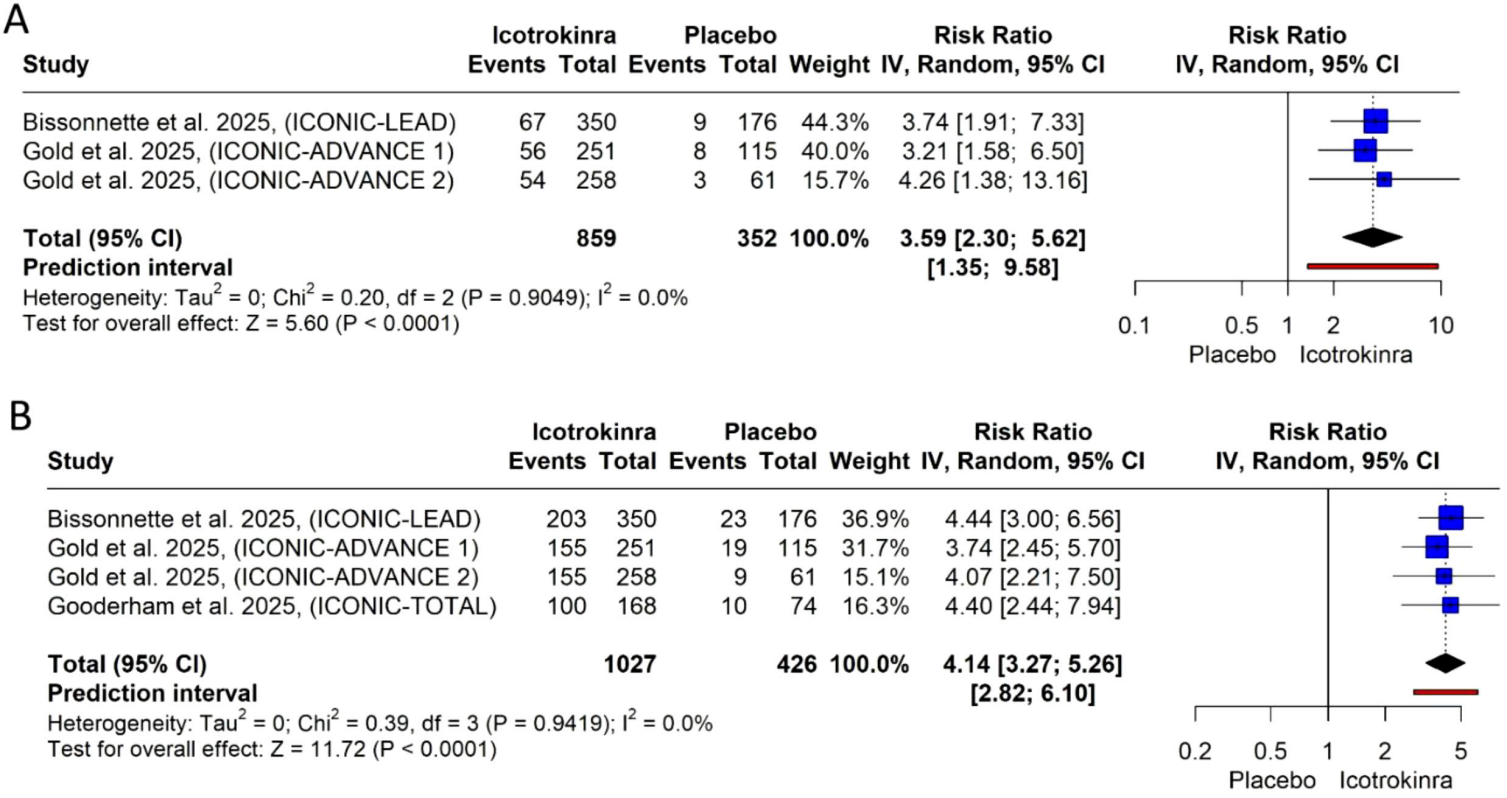
Forest plots for PSSD itch score: **(A)** for week 4 and **(B)** for week 16.

Clinically meaningful improvement in PSSD itch score at weeks 4 and 16: The pooled analysis demonstrated that icotrokinra significantly increased the PSSD itch score at weeks 4 and 16 compared to placebo [RR = 3.59, 95% CI (2.30; 5.62), *p* < 0.001; RR = 4.14, 95% CI (3.27; 5.26), *p* < 0.001, respectively) (**Figures 9A, B**). The pooled analysis was homogeneous, and *I*^2^ = 0 at weeks 4 and 16. At week 4, the LFK index was 0.55 (no asymmetry) (**Supplementary Figure 17**); however, at week 16, the LFK index was −1.79 (minor asymmetry) (**Supplementary Figure 18**). At week 16, the trim-and-fill analysis detected no evidence of publication bias, and the pooled estimate remained highly significant (**Supplementary Table 9**; **Supplementary Figure 19**).

#### 3.4.3 Safety outcomes: adverse events

**Figure 10:**
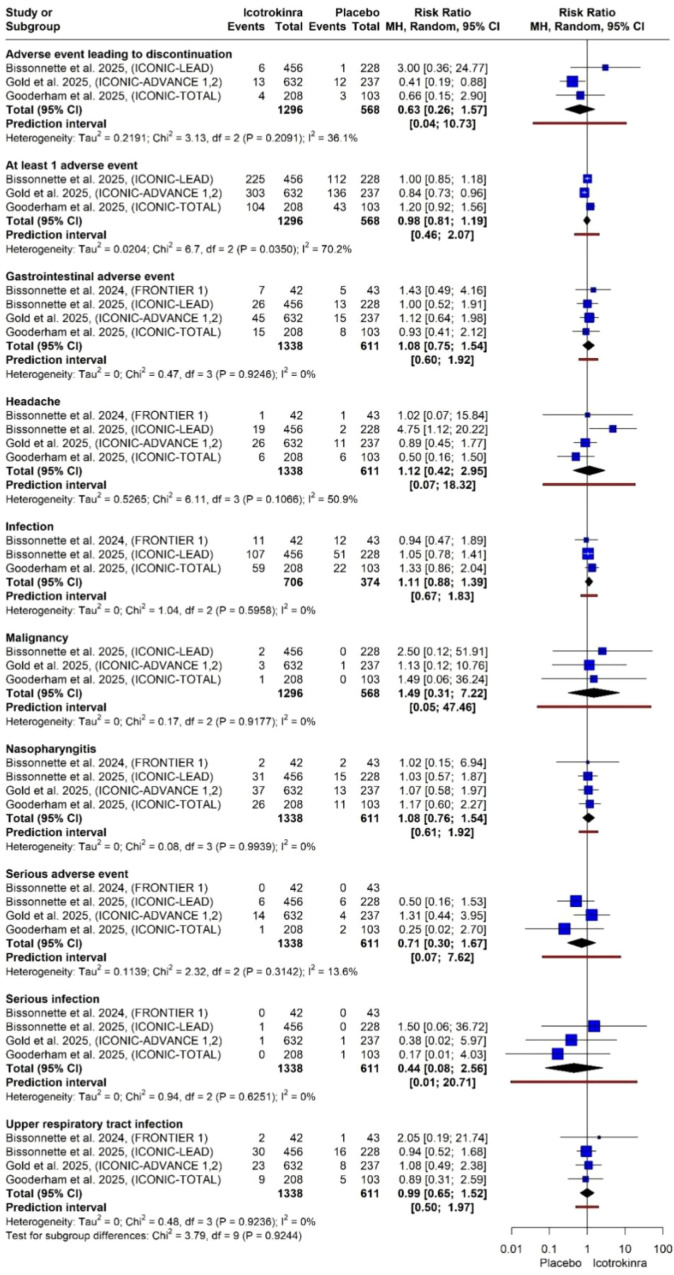
Forest plot for all adverse events.

Overall and serious adverse events: The risk of experiencing at least one adverse event was comparable between groups (RR 0.98, 95% CI 0.81–1.19). Similarly, icotrokinra was not associated with an increased risk of serious adverse events (RR 0.71, 95% CI 0.30–1.67) or adverse events leading to treatment discontinuation (RR 0.63, 95% CI 0.26–1.57) (**Figure 10**).

Infections and respiratory events: There was no significant increase in the risk of overall infection (RR 1.11, 95% CI 0.88–1.39). Analysis of the most frequent respiratory events showed no significant difference for nasopharyngitis (RR 1.08, 95% CI 0.76–1.54) or upper respiratory tract infections (RR 0.99, 95% CI 0.65–1.52). Notably, the risk for serious infections remained low and non-significant (RR 0.44, 95% CI 0.08–2.56) (**Figure 10**).

Other adverse events: No significant differences were observed for other systemic or localized events, including:

- gastrointestinal adverse events: RR 1.08 (95% CI 0.75–1.54).
- headache: RR 1.12 (95% CI 0.42–2.95).
- malignancy: RR 1.49 (95% CI 0.31–7.22).

Across most specific infection and systemic categories, heterogeneity was negligible (**Figure 10**).

#### 3.4.5 Trial sequential analysis

Based on the TSA findings, we found no conclusive evidence for any of the pre-specified efficacy outcomes. At week 16, the cumulative *Z*-curves for all endpoints, including PASI 75 (**Supplementary Figure 20**), PASI 90 (**Supplementary Figure 21**), PASI 100 (**Supplementary Figure 22**), ss-IGA (**Supplementary Figure 23**), PSSD symptom score of 0 (**Supplementary Figure 24**), and PSSD itch score (**Supplementary Figure 25**), remained below the trial sequential monitoring boundaries and the conventional significance boundary. Furthermore, the sample sizes for all analyses were significantly smaller than the required information sizes, which ranged from 3,270 to 10,186 individuals. This suggests that the current evidence is inadequate and inconclusive and that additional well-powered, large-scale RCTs are required to reach reliable results.

## 4 Discussion

### 4.1 Summary of findings

Our pooled analysis of 1,951 patients with moderate-to-severe plaque psoriasis indicated that patients treated with icotrokinra achieved a significantly higher rate of clear or almost clear skin (IGA 0/1) and completely clear skin (IGA 0) at week 16. This was consistently confirmed by significant PASI 75 achievement at week 16. Also, improvements of PASI 90 and 100 were significantly different from the placebo. For ss-IGA and PSSD, the icotrokinra group was observed to have a significant difference from placebo, and the incidence of adverse events did not differ significantly between the two groups.

### 4.2 Interpretation of findings

Moderate-to-severe plaque psoriasis is characterized by painful, pruritic lesions that profoundly burden patients’ quality of life. At week 16, patients receiving icotrokinra were nearly 28 times more likely to achieve completely clear skin (IGA 0) (27) and over 7 times more likely to achieve clear or almost clear status (IGA 0/1), compared with those on placebo.

In line with our findings, ICONIC-ADVANCE 1/2 (12), ICONIC-LEAD (13), ICONIC-TOTAL (11), and FRONTIER-1 (17) indicated that icotrokinra consistently demonstrated a marked superiority to placebo. For instance, the RR of our pooled analysis for achieving IGA 0 at week 16 with icotrokinra versus placebo was 24.11 [95% CI (12.31; 47.20), *p* < 0.001], and for IGA 0/1, the relative risk was 7.27 (95% CI 5.62–9.40). These results are highly consistent with the data reported across the pivotal trials: for instance, the ICONIC-LEAD (13) study reported IGA 0/1 rates of 65% for icotrokinra compared with 8% for placebo, and ICONIC-ADVANCE 1/2 (12) reported IGA 0/1 rates of 68%–70% for icotrokinra and 9%–11% for placebo at week 16. Additionally, the PASI 90 response rate at week 16 in the primary phase 3 studies ranged from approximately 50% to 57%, closely similar to the overall effect estimates derived from the pooled analysis (11–13).

The FRONTIER-2 study (16) provided long-term follow-up data at 52 weeks and with over 89% of participants from the original 16-week trial (FRONTIER-1) (17) continued into FRONTIER-2. Achieved improvements in efficacy outcomes at week 16 were sustained through week 52, with patients receiving a 200 mg daily dose showing maintenance of a PASI 75 response, PASI 90, PASI 100, and IGA 0/1 (16). Furthermore, PSSD itch scores maintained significant improvement at long-term follow-up with consistent safety. In FRONTIER-2, 59% of patients experienced at least one adverse event over 1 year, but no dose-dependent increase in adverse events or safety signals was observed. The incidence rates of serious adverse events were uncommon and not related to the trial intervention. Improvements in difficult-to-treat areas, such as the scalp, were significant and sustained for 52 weeks (16).

The most recent comprehensive Cochrane network meta-analysis (179 trials; 62,000 patients) confirmed that biologics remain more effective at achieving PASI 90 than non-biologic systemics and small molecules such as apremilast and deucravacitinib (28). Tofacitinib and fumaric acid esters, available outside the USA, also show moderate efficacy and carry concerns regarding safety and regulatory approval status (5, 29).

The pooled PASI 75 and PASI 90 response rates in our analysis are markedly higher than the PASI 75 rates reported in trials of apremilast and exceed the PASI 75 and PASI 90 rates observed with deucravacitinib (5, 29–31). Although icotrokinra does not match the highest PASI 90 responses achieved by agents like infliximab, bimekizumab, ixekizumab, or risankizumab, its efficacy is comparable to that of several IL inhibitor biologics (28). This perspective might be challenging to the long-standing view that oral therapies are inherently less effective than injectable biologics (3, 32, 33).

Furthermore, our synthesis builds upon earlier network meta-analysis (NMA), such as Sbidian et al. (34), which established the superiority of biologics over the oral agents available at the time. The ICONIC-ADVANCE 1/2 trials added important further clarity by providing direct, head-to-head comparisons demonstrating that icotrokinra outperformed deucravacitinib consistently in PASI 90, IGA 0/1, and site-specific endpoints (12).

An NMA by Egeberg et al. reported that IL-17 inhibitors may achieve PASI 90 responses more rapidly than injectable IL-23 inhibitors (35). Future research should clarify where icotrokinra falls within this timing spectrum. Oral systemic agents have been associated with safety concerns. Methotrexate carries risks of hepatotoxicity and hematological suppression, cyclosporine is limited to short-term use due to nephrotoxicity and hypertension, and acitretin is constrained by teratogenicity and mucocutaneous side effects (36–38). In our study, icotrokinra showed a safety and tolerability profile similar to placebo. Most adverse events were mild infections, such as nasopharyngitis and URTIs. This pattern is in line with the safety experience of injectable IL-23 inhibitors, which have been found to have a tolerated safety profile, with no increased rates of serious infections or malignancies (3). The Cochrane NMA likewise found no meaningful differences in serious adverse events between most active treatments and placebo (28). Nonetheless, our findings, like prior studies of other interventions, remain limited by the relatively short induction periods of available RCTs and a lack of robust long-term extension or real-world cohort data for the novel oral agents (28, 29).

### 4.3 Strengths and limitations

This study, to the best of our knowledge, is the first meta-analysis to assess the pooled safety and efficacy of icotrokinra in patients with plaque psoriasis. Our findings were based on a rigorous methodology, incorporating pooled data from high-quality, placebo-controlled multicenter RCTs with consistent results and overall low risk of bias. However, this study has limitations that should be considered when interpreting the findings. The analysis is based on a small number of manufacturer-sponsored trials. They enrolled homogeneous patients, with exclusion of those with complex comorbidities or treatment-refractory disease. This likely contributed to low observed heterogeneity but restricts broader application in real-world clinical settings. Although the trim-and-fill method, used to adjust for this asymmetry found in funnel plots of IGA and PASI outcomes, confirmed that the results remained highly significant, this finding suggests a potential for publication bias or small-study effects. The underreporting of unfavorable data cannot be fully excluded. Notably, the TSA demonstrated that the required information size has not been reached for any efficacy outcome. This indicates that the available evidence remains insufficient and not reliable to draw definitive conclusions about treatment effects, despite the promising point estimates and narrow confidence intervals. The safety profile of the drug, while reassuring, is therefore limited by the short follow-up duration of the included trials (primarily 16 weeks) and the absence of long-term extension data in the pooled analysis.

### 4.4 Clinical implications and future recommendations

The findings from individually included RCTs, in addition to the findings of our meta-analysis, indicate that oral icotrokinra could be considered an effective and well-tolerated treatment option for moderate-to-severe plaque psoriasis patients who are candidates for systemic therapy. The AAD guidelines position apremilast as suitable for patients willing to “accept a slower onset of skin clearance and slower likelihood of clearing” in exchange for avoiding frequent injections and laboratory monitoring (3, 5). However, given its favorable efficacy profile, with PASI 90 and IGA 0/1 response rates comparable to those of certain injectable biologics, icotrokinra could particularly be recommended for patients who are not compliant with injections or have contraindications to existing systemic agents. The favorable safety profile found in our pooled analysis further supports its use.

Future, powered, long-term RCTs and studies are particularly needed to evaluate the maintenance of efficacy and to monitor for any potential adverse events associated with continuous use. Direct head-to-head RCTs will also be important to define icotrokinra’s position within the therapeutic hierarchy, including comparisons with other modern oral agents (apremilast) and with parenteral biologics. For a clearer picture of its role in routine clinical practice, real-world evidence studies are needed. This can confirm the effectiveness and safety of icotrokinra in broader, more diverse populations, including patients with comorbidities and prior treatment exposures who are often excluded from currently available RCTs.

## 5 Conclusion

Our meta-analysis demonstrated that icotrokinra has promising preliminary efficacy and is well-tolerated for moderate-to-severe plaque psoriasis. Icotrokinra can significantly improve IGA and PASI scores, as well as PSSD scores. Its clinical utility is supported by its favorable safety profile. However, TSA indicates that the present evidence is insufficient. While icotrokinra may represent a potential therapeutic advancement offering biologic-like efficacy in an oral formulation, the current evidence remains insufficient to draw definitive conclusions. Larger, well-powered RCTs are needed to validate these findings and provide long-term follow-up.

## Data Availability

The original contributions presented in the study are included in the article/**Supplementary Material**. Further inquiries can be directed to the corresponding author.
