# Supplementary material for: Safety and efficacy of oral icotrokinra for moderate-to-severe plaque psoriasis: a systematic review and meta-analysis of randomized controlled trials
Source: Front Immunol. 2026 Mar 6;17:1768292. doi: 10.3389/fimmu.2026.1768292 (PMC13002440; doi:10.3389/fimmu.2026.1768292)
Supplement: Supplementary file 1 [file Table1.docx]

**Supplementary material**

| **Index of Contents** | **Page** |
| --- | --- |
| **TABLES** |  |
| **Table S1.** Systematic Search Strategy and Results. | 3 |
| **Table S2.** Trim-and-fill analysis for Investigator's Global Assessment (IGA) 0 response at week 16. | 4 |
| **Table S3.** Trim-and-fill analysis for PASI 75 response at week 16. | 5 |
| **Table S4.** Trim-and-fill analysis for PASI 90 response at week 16. | 6 |
| **Table S5.** Trim-and-fill analysis for PASI 100 response at week 16. | 7 |
| **Table S6.** Trim-and-fill analysis for Scalp-specific Investigator's Global Assessment (ss-IGA) 0/1 response at week 16. | 8 |
| **Table S7.** Trim-and-fill analysis for Psoriasis Symptom Scale Diary (PSSD) symptom score of 0 at week 8. | 9 |
| **Table S8.** Trim-and-fill analysis for PSSD symptom score of 0 at week 16. | 10 |
| **Table S9.** Trim-and-fill analysis for clinically meaningful improvement in PSSD itch score at week 16. | 11 |
| **FIGURES** |  |
| **Figure S1.** Doi plot for IGA response (IGA 0/1) at week 16. | 12 |
| **Figure S2.** Doi plot for IGA response (IGA 0) at week 16. | 13 |
| **Figure S3.** Funnel plot after trim and fill for IGA response (IGA 0) at week 16. | 14 |
| **Figure S4.** Doi plot for PASI 75 response at week 4. | 15 |
| **Figure S5.** Doi plot for PASI 75 response at week 16. | 16 |
| **Figure S6.** Funnel plot after trim and fill for PASI 75 response at week 16. | 17 |
| **Figure S7.** Doi plot for PASI 90 response at week 16. | 18 |
| **Figure S8.** Funnel plot after trim and fill for PASI 90 response at week 16. | 19 |
| **Figure S9.** Doi plot for PASI 100 response at week 16. | 20 |
| **Figure S10.** Funnel plot after trim and fill for PASI 100 response at week 16. | 21 |
| **Figure S11.** Doi plot for Area Specific Improvement (ss-IGA). | 22 |
| **Figure S12.** Funnel plot after trim and fill for Area Specific Improvement (ss-IGA). | 23 |
| **Figure S13.** Doi plot for PSSD symptom score of 0 at week 8. | 24 |
| **Figure S14.** Doi plot for PSSD symptom score of 0 at week 16. | 25 |
| **Figure S15.** Funnel plot after trim and fill for PSSD symptom score of 0 at week 8. | 26 |
| **Figure S16.** Funnel plot after trim and fill for PSSD symptom score of 0 at week 16. | 27 |
| **Figure S17.** Doi plot for PSSD itch score at week 4. | 28 |
| **Figure S18.** Doi plot for PSSD itch score at week 16. | 29 |
| **Figure S19.** Funnel plot after trim and fill for PSSD itch score at week 16. | 30 |
| **Figure S20.** Trial Sequential Analysis (TSA) for PASI 75 response at week 16. | 31 |
| **Figure S21.** Trial Sequential Analysis (TSA) for PASI 90 response at week 16. | 31 |
| **Figure S22.** Trial Sequential Analysis (TSA) for PASI 100 response at week 16. | 32 |
| **Figure S23.** Trial Sequential Analysis (TSA) for area-specific improvement at week 16. | 32 |
| **Figure S24.** Trial Sequential Analysis (TSA) for Scalp-specific Investigator's Global Assessment (ss-IGA) 0/1 response at week 16. | 33 |
| **Figure S25.** Trial Sequential Analysis (TSA) for a clinically meaningful improvement in Psoriasis Symptom Scale Diary (PSSD) itch score at week 16. | 33 |
| **PRISMA Checklist** | 34-35 |

| Databases | Search strategies | Results | Limitations | Date of search |
| --- | --- | --- | --- | --- |
| PubMed | All Fields (Icotrokinra OR "IL-23 receptor antagonist" OR JNJ-2113 OR JNJ-77242113) AND ("Plaque Psoriasis"OR "Psoriasis Vulgaris") | 7 | No limitations applied | 9 November 2025 |
| Cochrane Library | (Icotrokinra OR IL-23 receptor antagonist OR JNJ-2113 OR JNJ-77242113) AND (Plaque Psoriasis OR Psoriasis Vulgaris) in All Text | 40 | No limitations applied | 9 November 2025 |
| Scopus | ALL ( ( Icotrokinra OR "IL-23 receptor antagonist" OR JNJ-2113 OR JNJ-77242113) AND ( "Plaque Psoriasis" OR "Psoriasis Vulgaris" ) ) | 48 | No limitations applied | 9 November 2025 |
| Web of Science | (Icotrokinra OR IL-23 receptor antagonist OR JNJ-2113 OR JNJ-77242113) AND (Plaque Psoriasis OR Psoriasis Vulgaris) All Fields | 40 | No limitations applied | 9 November 2025 |

Table S1. Search strategies.

| Study | RR | 95% CI | % Weight (Random) |
| --- | --- | --- | --- |
| Bissonnette et al., 2024 (FRONTIER-1) | 39.92 | [2.49 – 640.29] | 5 |
| Bissonnette et al., 2025 (ICONIC-LEAD) | 25.33 | [8.17 – 78.55] | 30 |
| Gold et al., 2025 (ICONIC-ADVANCE 1) | 27.86 | [8.97 – 86.55] | 29.9 |
| Gold et al., 2025 (ICONIC-ADVANCE 2) | 30.05 | [4.26 – 211.90] | 10.1 |
| Gooderham et al., 2025 (ICONIC-TOTAL) | 26.25 | [3.68 – 187.11] | 10 |
| Filled: Gold et al., 2025 (ICONIC-ADVANCE 2) | **23.4** | **[3.32 – 165.02]** | **10.1** |
| Filled: Bissonnette et al., 2024 (FRONTIER-1) | **17.62** | **[1.10 – 282.57]** | **5** |
| Pooled (Random-Effects Model) | **26.52** | **[14.27 – 49.29]** | 100 |
| Heterogeneity | |  |  |
| τ² = 0; I² = 0.0% [0.0–70.8%]; Q = 0.21, p = 0.9998 | | | |
| Trim-and-Fill Details | |  |  |
| Added (filled) studies = 2 | | |  |
| Adjustment method = L-estimator | | | |
| Publication bias effect = Minimal; adjusted RR remains highly significant | | | |

Table S2. Trim-and-fill analysis for IGA 0 response at week 16.

| Study | RR | 95% CI | % Weight (Random) |
| --- | --- | --- | --- |
| Bissonnette et al., 2024 (FRONTIER-1) | 8.45 | [3.28 – 21.77] | 6.6 |
| Bissonnette et al., 2025 (ICONIC-LEAD) | 6.3 | [4.33 – 9.17] | 42.3 |
| Gold et al., 2025 (ICONIC-ADVANCE 1) | 6.44 | [4.15 – 9.99] | 30.8 |
| Gold et al., 2025 (ICONIC-ADVANCE 2) | 7.93 | [4.09 – 15.35] | 13.6 |
| Filled: Bissonnette et al., 2024 (FRONTIER-1) | 5.13 | [1.99 – 13.21] | 6.6 |
| Pooled (Random-Effects Model) | 6.58 | [5.16 – 8.40] | 100 |
| Heterogeneity | |  |  |
| τ² = 0 [0.0000–0.1348]; I² = 0.0% [0.0–79.2%]; Q = 0.90, p = 0.9246 | | | |
| Trim-and-Fill Details | |  |  |
| Added (filled) studies = 1 | | |  |
| Adjustment method = L-estimator | | | |
| Publication bias effect = Minimal; adjusted RR remains highly significant | | | |

Table S3. Trim-and-fill analysis for PASI 75 response at week 16.

| Study | RR | 95% CI | % Weight (Random) |
| --- | --- | --- | --- |
| Bissonnette et al., 2024 (FRONTIER-1) | 25.6 | [3.63 – 180.45] | 4.9 |
| Bissonnette et al., 2025 (ICONIC-LEAD) | 11.3 | [6.12 – 20.86] | 50.1 |
| Gold et al., 2025 (ICONIC-ADVANCE 1) | 14.3 | [6.48 – 31.53] | 30.1 |
| Gold et al., 2025 (ICONIC-ADVANCE 2) | 46.86 | [6.66 – 329.43] | 5 |
| Filled: Bissonnette et al., 2024 (FRONTIER-1) | 5.95 | [0.84 – 41.96] | 4.9 |
| Filled: Gold et al., 2025 (ICONIC-ADVANCE 2) | 3.25 | [0.46 – 22.86] | 5 |
| Pooled (Random-Effects Model) | 12.34 | [7.99 – 19.05] | 100 |
| Heterogeneity | |  |  |
| τ² < 0.0001 [0.0000–4.6121]; τ = 0.0008 [0.0000–2.1476]; I² = 0.0% [0.0–74.6%]; Q = 4.88, p = 0.4309 | | | |
| Trim-and-Fill Details | |  |  |
| Added (filled) studies = 2 | | |  |
| Adjustment method = L-estimator | | | |
| Publication bias effect = Minimal; adjusted RR remains highly significant | | | |

Table S4. Trim-and-fill analysis for PASI 90 response at week 16.

| Study | RR | 95% CI | % Weight (Random) |
| --- | --- | --- | --- |
| Bissonnette et al., 2024 (FRONTIER-1) | 35.82 | [2.22 – 576.96] | 11.1 |
| Bissonnette et al., 2025 (ICONIC-LEAD) | 61.5 | [8.65 – 437.26] | 22.2 |
| Gold et al., 2025 (ICONIC-ADVANCE 1) | 24.33 | [6.08 – 97.37] | 44.4 |
| Gold et al., 2025 (ICONIC-ADVANCE 2) | 25.98 | [3.68 – 183.41] | 22.4 |
| Pooled (Random-Effects Model) | 31.65 | [12.56 – 79.76] | 100 |
| Heterogeneity | |  |  |
| τ² = 0 [0.0000–1.6111]; τ = 0 [0.0000–1.2693]; I² = 0.0% [0.0–84.7%]; Q = 0.63, p = 0.8905 | | | |
| Trim-and-Fill Details | |  |  |
| Added (filled) studies = 0 | | |  |
| Adjustment method = L-estimator | | | |
| Publication bias effect = Not detected; pooled RR remains highly significant | | | |

Table S5. Trim-and-fill analysis for PASI 100 response at week 16.

| Study | RR | 95% CI | % Weight (Random) |
| --- | --- | --- | --- |
| Bissonnette et al., 2025 (ICONIC-LEAD) | 4.84 | [3.46 – 6.77] | 30.6 |
| Gold et al., 2025 (ICONIC-ADVANCE 1) | 3.47 | [2.47 – 4.86] | 30.3 |
| Gold et al., 2025 (ICONIC-ADVANCE 2) | 3.9 | [2.37 – 6.40] | 16.7 |
| Gooderham et al., 2025 (ICONIC-TOTAL) | 6.22 | [3.32 – 11.65] | 11.2 |
| Filled: Gooderham et al., 2025 (ICONIC-TOTAL) | 2.65 | [1.42 – 4.97] | 11.2 |
| Pooled (Random-Effects Model) | 4.06 | [3.25 – 5.07] | 100 |
| Heterogeneity | |  |  |
| τ² = 0.0127 [0.0000–0.7775]; τ = 0.1125 [0.0000–0.882]; I² = 26.9% [0.0–71.1%]; Q = 5.47, p = 0.2421 | | | |
| Trim-and-Fill Details | |  |  |
| Added (filled) studies = 1 | | |  |
| Adjustment method = L-estimator | | | |
| Publication bias effect = Minimal; pooled RR remains highly significant | | | |

Table S6. Trim-and-fill analysis for ss-IGA 0/1 response at week 16.

| Study | RR | 95% CI | % Weight (Random) |
| --- | --- | --- | --- |
| Bissonnette et al., 2025 (ICONIC-LEAD) | 4.93 | [1.52 – 15.99] | 37.1 |
| Gold et al., 2025 (ICONIC-ADVANCE 1) | 3.97 | [1.22 – 12.97] | 36.7 |
| Gold et al., 2025 (ICONIC-ADVANCE 2) | 6.43 | [0.89 – 46.55] | 13.1 |
| Filled: Gold et al., 2025 (ICONIC-ADVANCE 2) | 3.05 | [0.42 – 22.04] | 13.1 |
| Pooled (Random-Effects Model) | 4.43 | [2.16 – 9.06] | 100 |
| Heterogeneity | |  |  |
| τ² = 0 [0.0000–0.4680]; I² = 0.0% [0.0–84.7%]; Q = 0.34, p = 0.953 | | | |
| Trim-and-Fill Details | |  |  |
| Added (filled) studies = 1 | | |  |
| Adjustment method = L-estimator | | | |
| Publication bias effect = Minimal; adjusted RR remains significant | | | |

Table S7. Trim-and-fill analysis for PSSD symptom score of 0 at week 8.

| Study | RR | 95% CI | % Weight (Random) |
| --- | --- | --- | --- |
| Bissonnette et al., 2024 (FRONTIER 1) | 23.54 | [1.43 – 387.01] | 4.6 |
| Bissonnette et al., 2025 (ICONIC-LEAD) | 20.9 | [5.19 – 84.15] | 18.4 |
| Gold et al., 2025 (ICONIC-ADVANCE 1) | 8.44 | [3.14 – 22.67] | 36.6 |
| Gold et al., 2025 (ICONIC-ADVANCE 2) | 30.9 | [1.94 – 493.38] | 4.7 |
| Gooderham et al., 2025 (ICONIC-TOTAL) | 4.71 | [1.48 – 14.98] | 26.6 |
| Filled: Bissonnette et al., 2024 (FRONTIER 1) | 3.11 | [0.19 – 51.15] | 4.6 |
| Filled: Gold et al., 2025 (ICONIC-ADVANCE 2) | 2.37 | [0.15 – 37.85] | 4.7 |
| Pooled (Random-Effects Model) | 8.56 | [4.71 – 15.55] | 100 |
| Heterogeneity | |  |  |
| τ² = 0 [0.0000–3.5862]; I² = 0.0% [0.0–70.8%]; Q = 5.26, p = 0.511 | | | |
| Trim-and-Fill Details | |  |  |
| Added (filled) studies = 2 | | |  |
| Adjustment method = L-estimator | | | |
| Publication bias effect = Minimal; adjusted RR remains significant | | | |

Table S8. Trim-and-fill analysis for PSSD symptom score of 0 at week 16.

| Study | RR | 95% CI | % Weight (Random) |
| --- | --- | --- | --- |
| Bissonnette et al., 2025 (ICONIC-LEAD) | 4.44 | [3.00 – 6.56] | 36.9 |
| Gold et al., 2025 (ICONIC-ADVANCE 1) | 3.74 | [2.45 – 5.70] | 31.7 |
| Gold et al., 2025 (ICONIC-ADVANCE 2) | 4.07 | [2.21 – 7.50] | 15.1 |
| Gooderham et al., 2025 (ICONIC-TOTAL) | 4.4 | [2.44 – 7.94] | 16.3 |
| Pooled (Random-Effects Model) | 4.14 | [3.27 – 5.26] | 100 |
| Heterogeneity | |  |  |
| τ² = 0 [0.0000–0.0388]; I² = 0.0% [0.0–84.7%]; Q = 0.39, p = 0.942 | | | |
| Trim-and-Fill Details | |  |  |
| Added (filled) studies = 0 | | |  |
| Adjustment method = L-estimator | | | |
| Publication bias effect = Minimal; pooled RR remains highly significant | | | |

Table S9. Trim-and-fill analysis for clinically meaningful improvement in PSSD itch score at week 16.


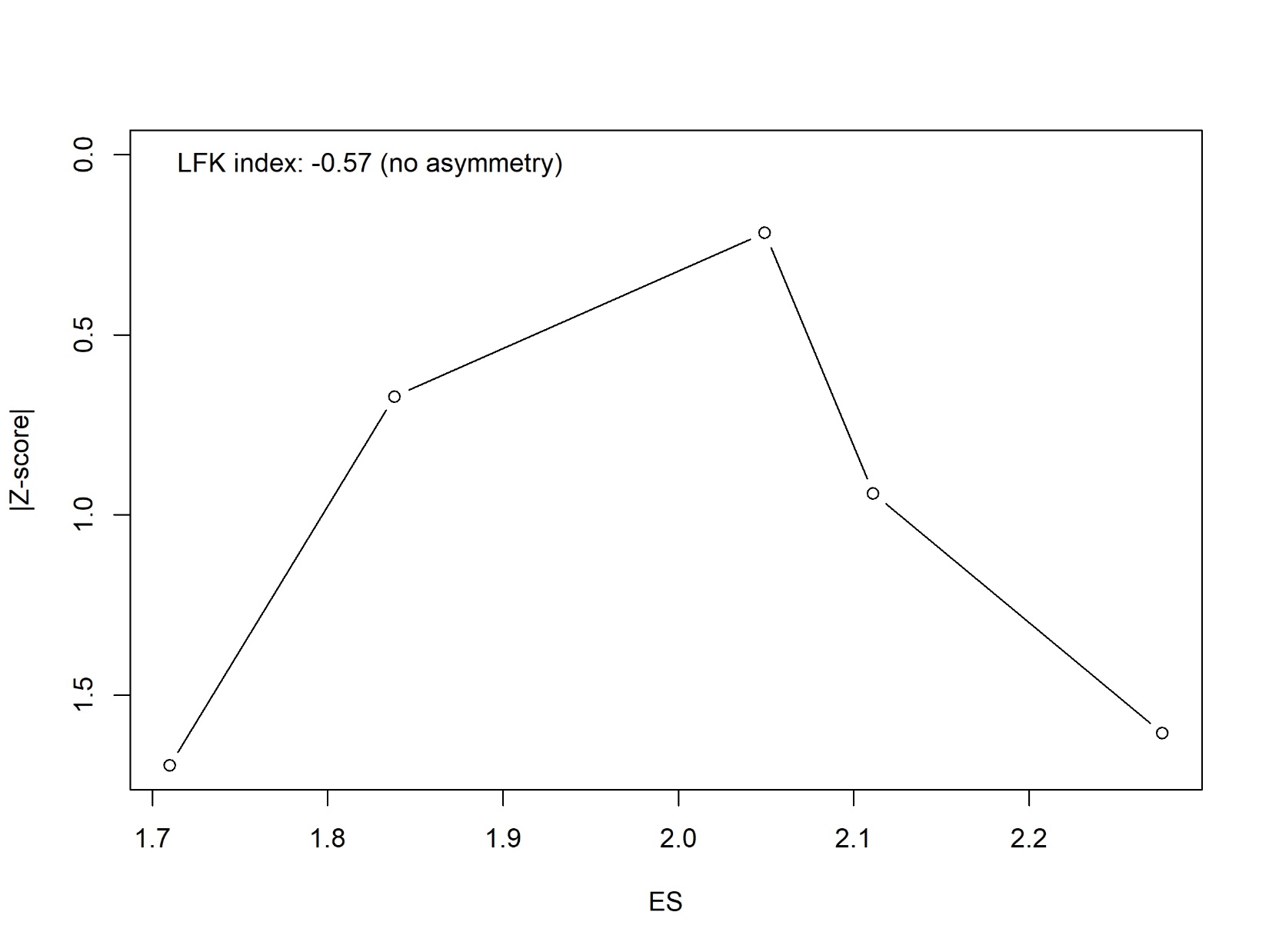


Figure S1: Doi plot for IGA response (IGA 0/1) at week 16


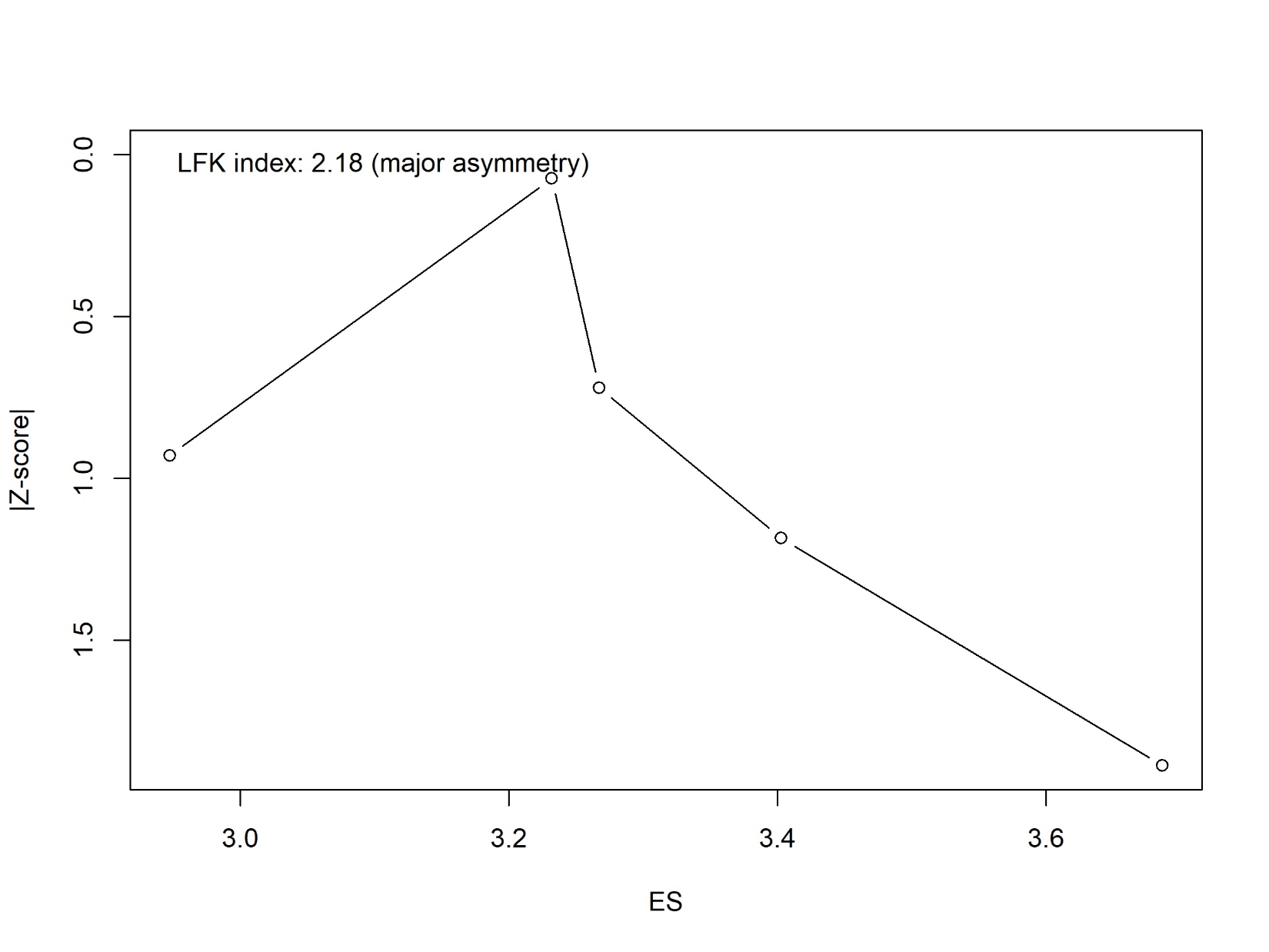


Figure S2: Doi plot for IGA response (IGA 0) at week 16


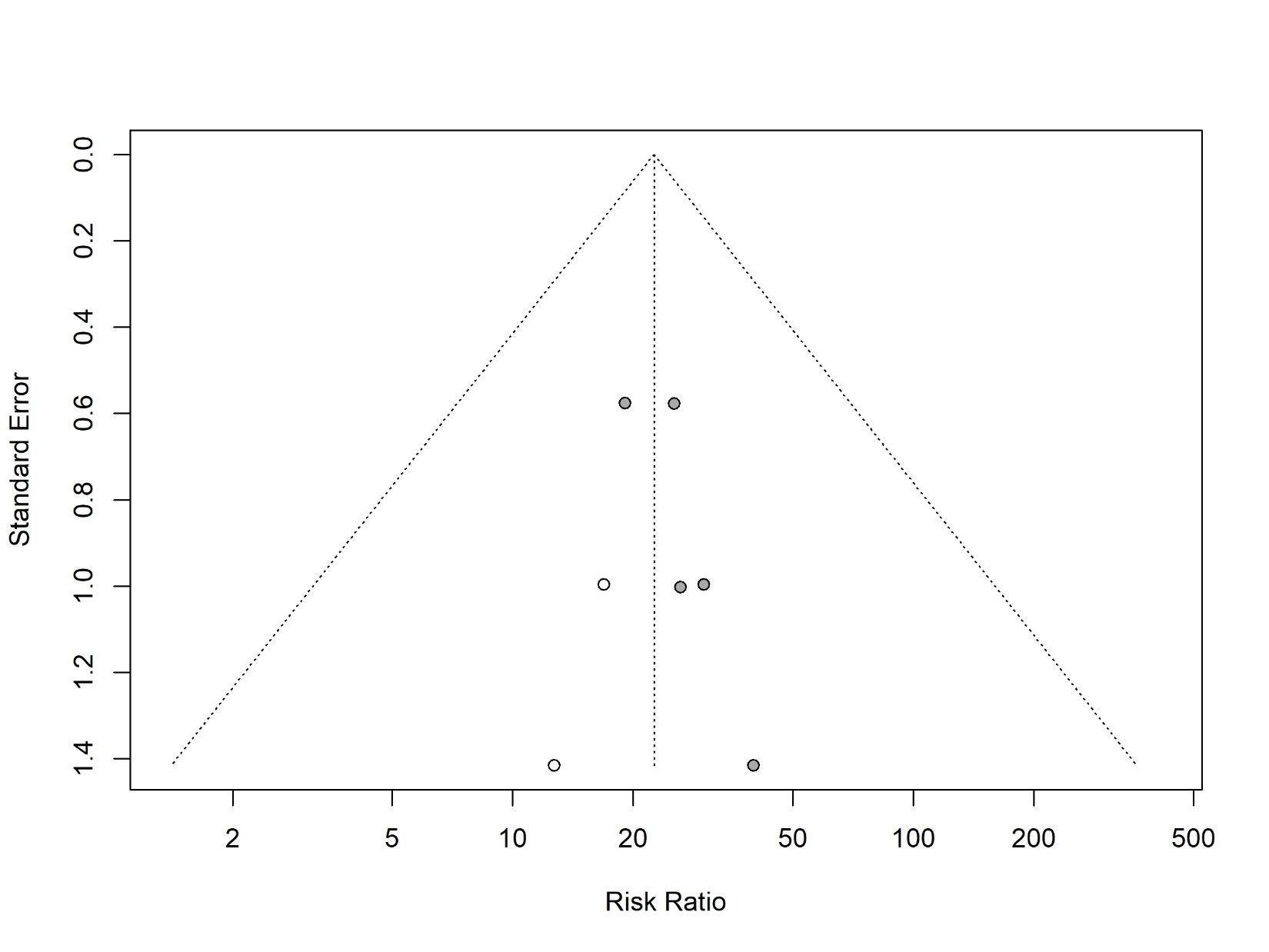


Figure S3: Funnel plot after trim and fill for IGA response (IGA 0) at week 16


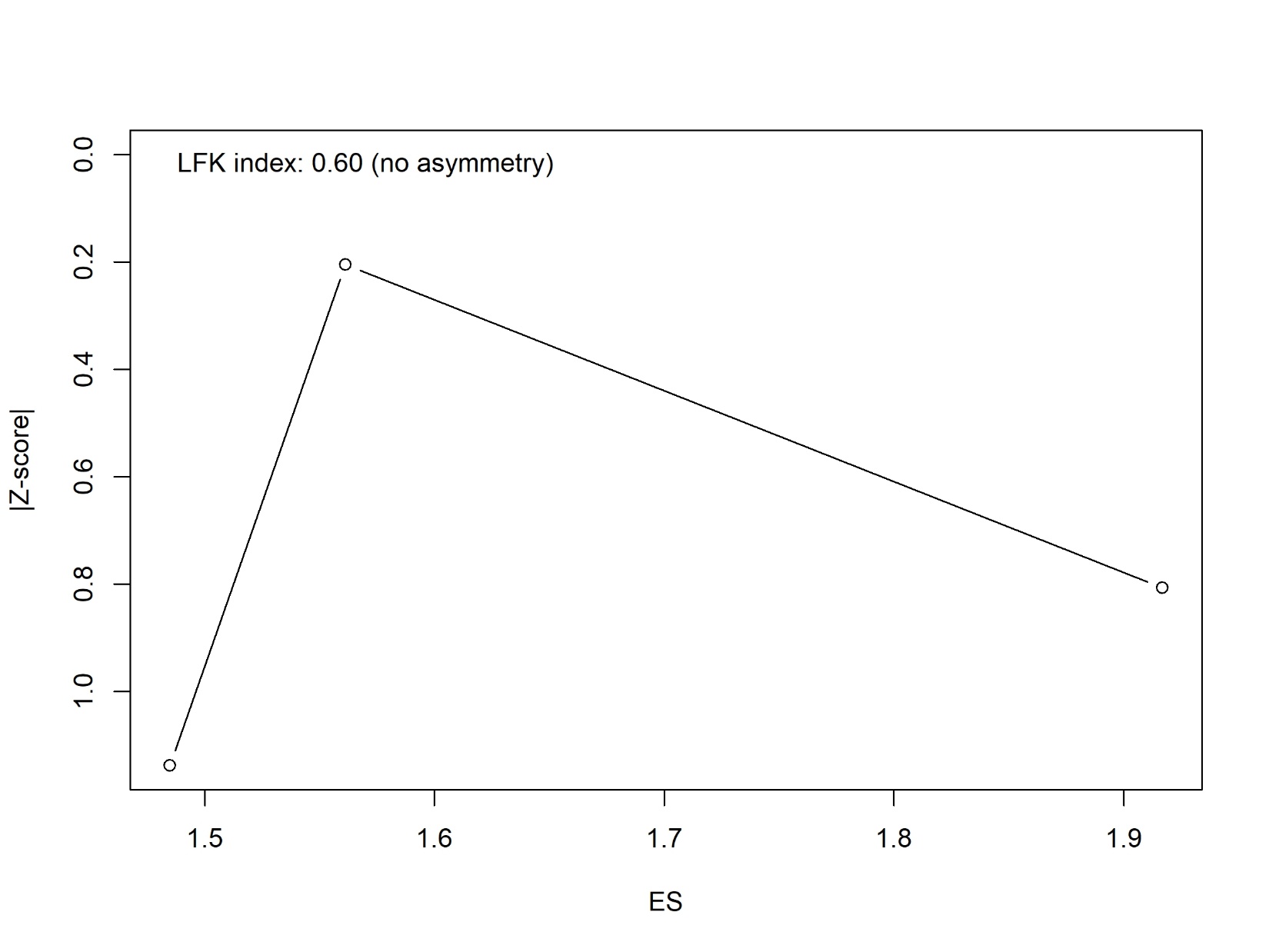


Figure S4: Doi plot for PASI 75 response at week 4


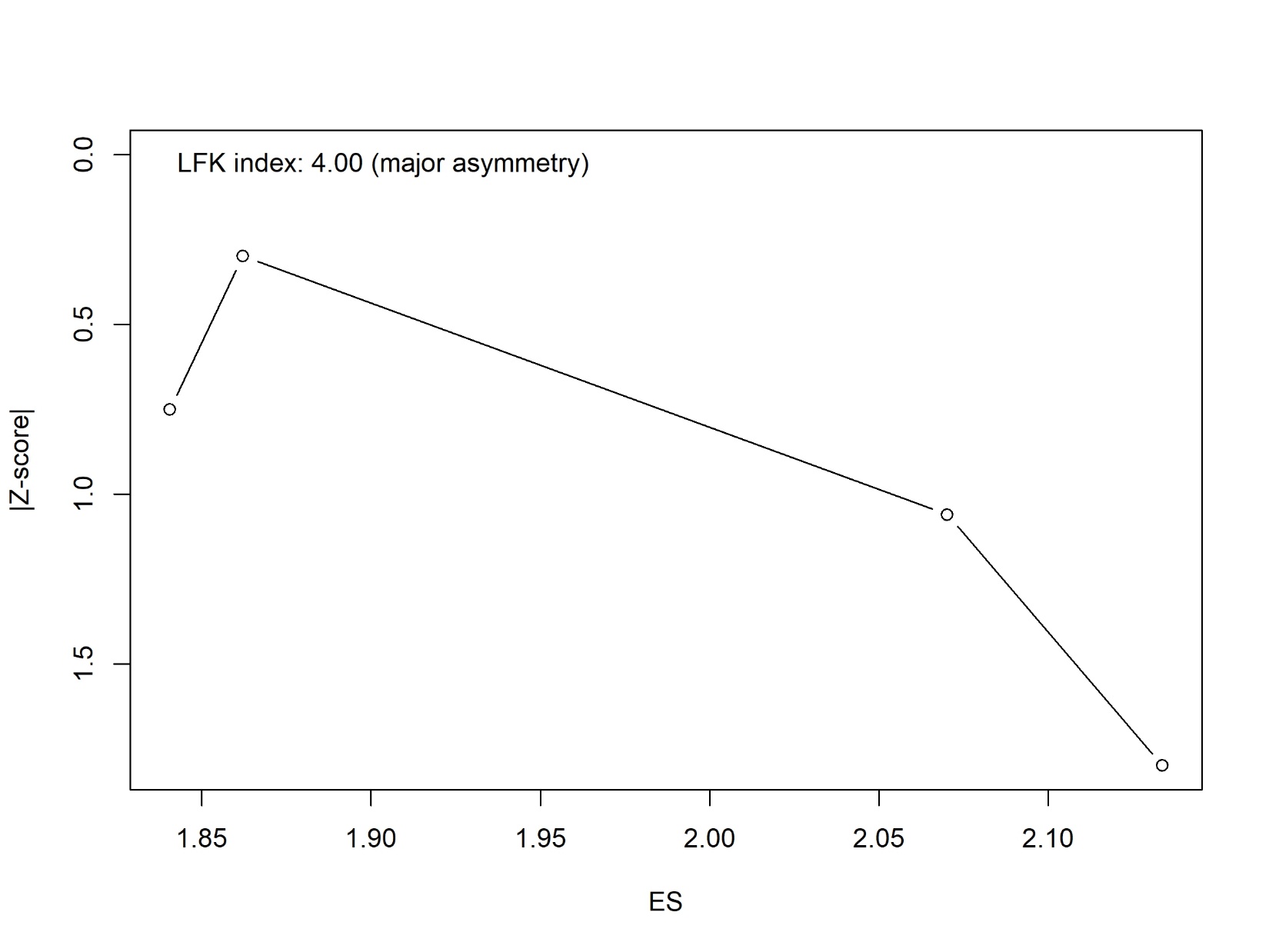


Figure S5: Doi plot for PASI 75 response at week 16


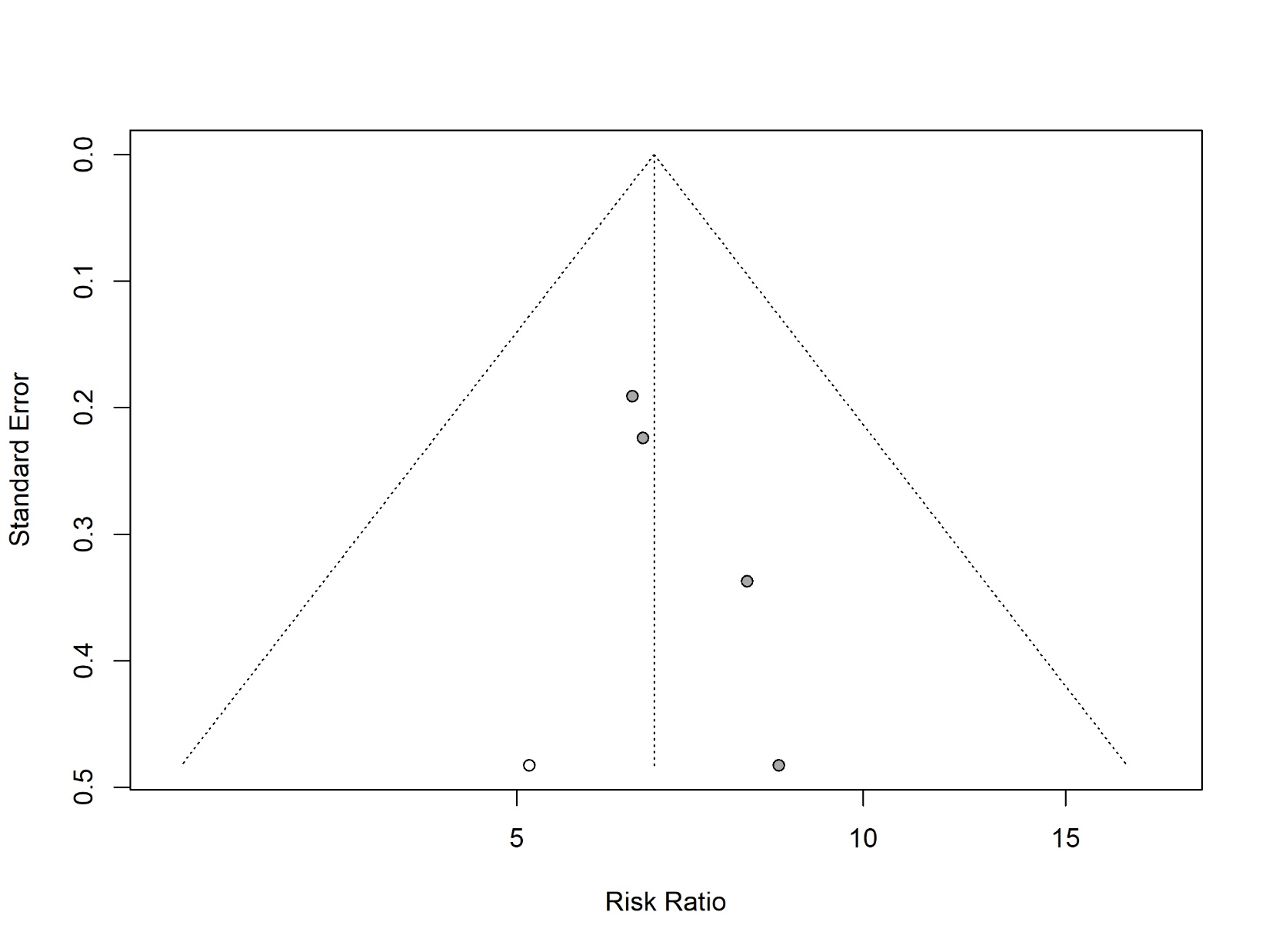


Figure S6: Funnel plot after trim and fill for PASI 75 response at week 16


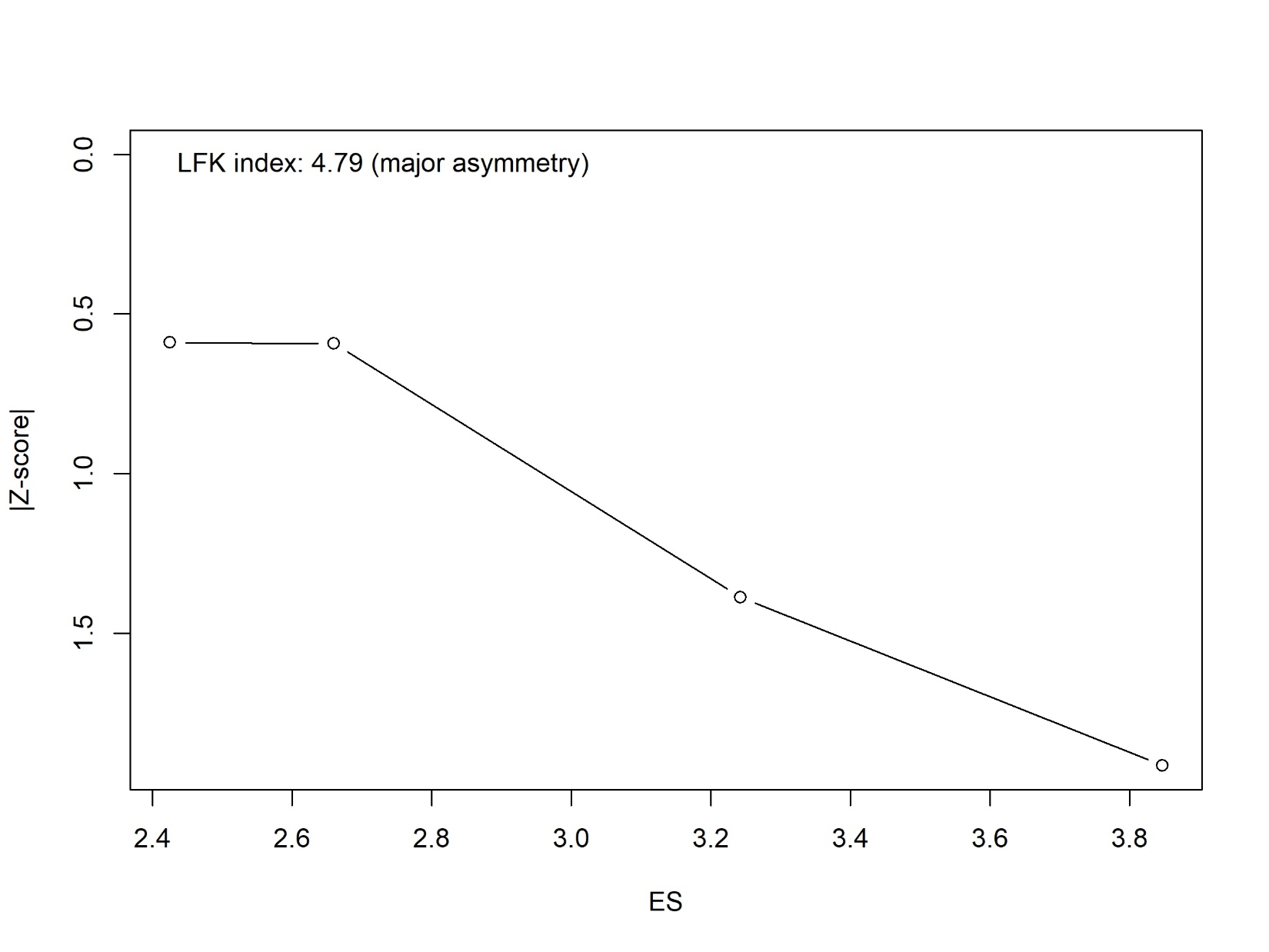


Figure S7: Doi plot for PASI 90 response at week 16


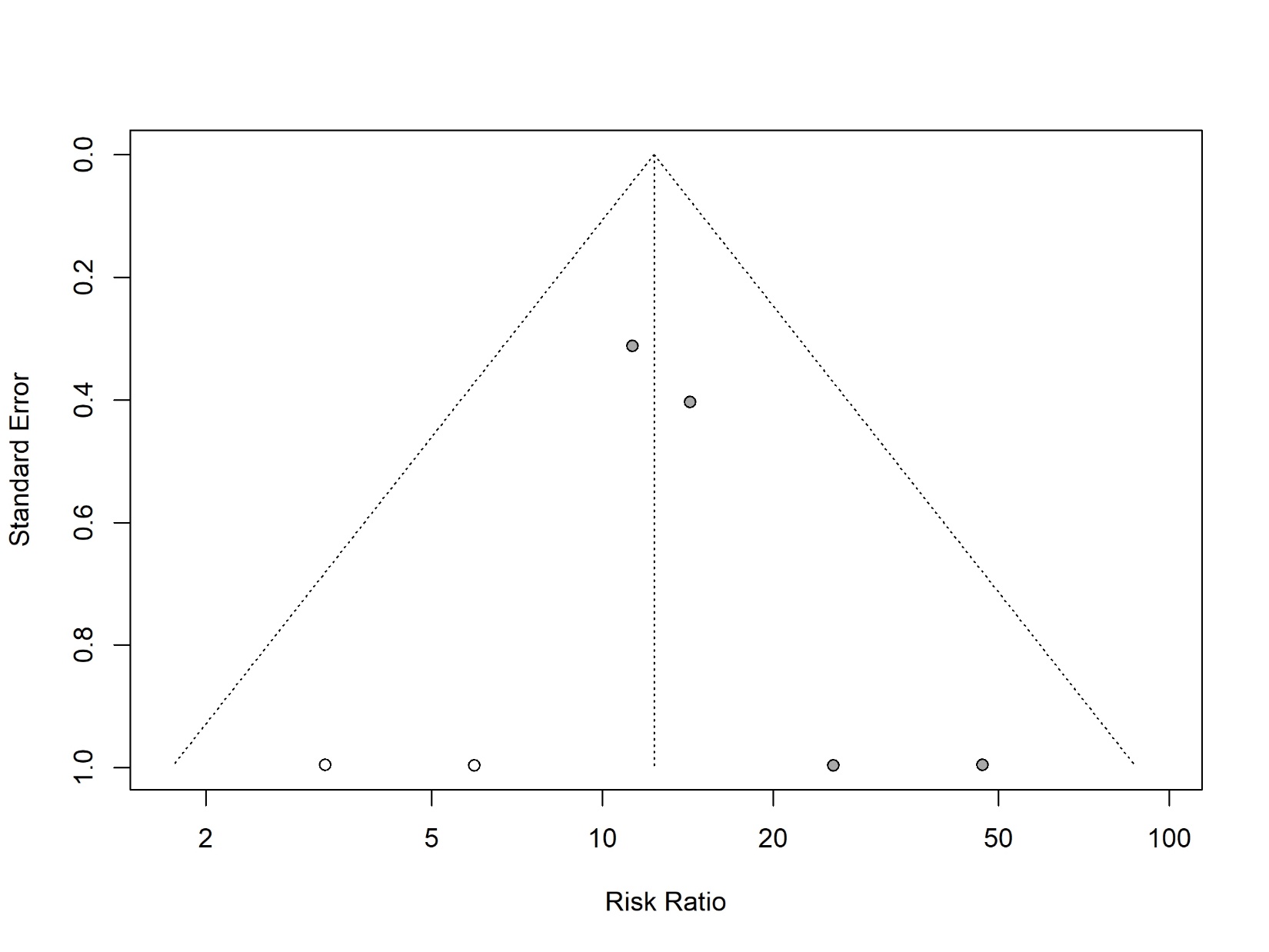


Figure S8: Funnel plot after trim and fill for PASI 90 response at week 16


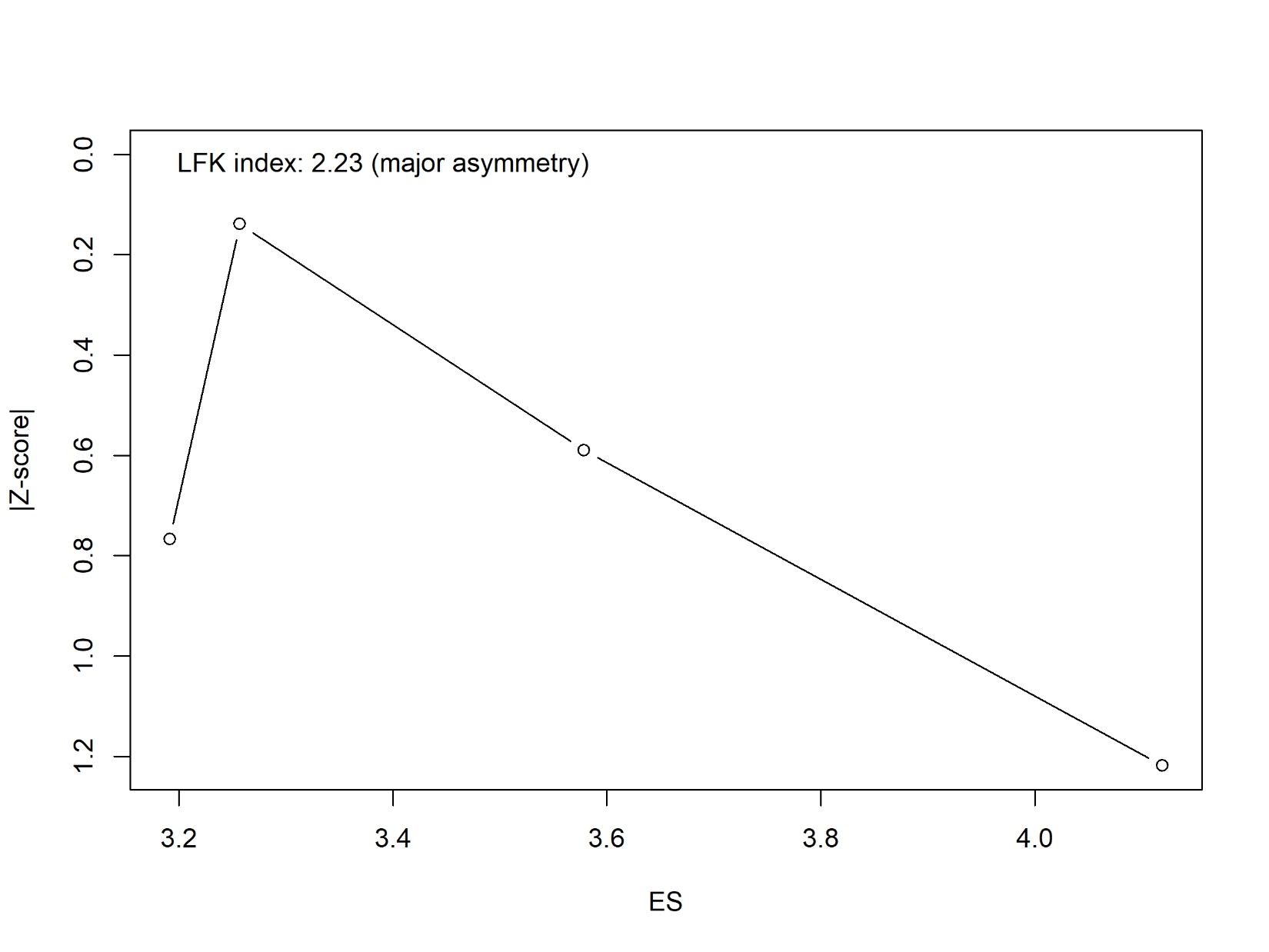


Figure S9: Doi plot for PASI 100 response at week 16


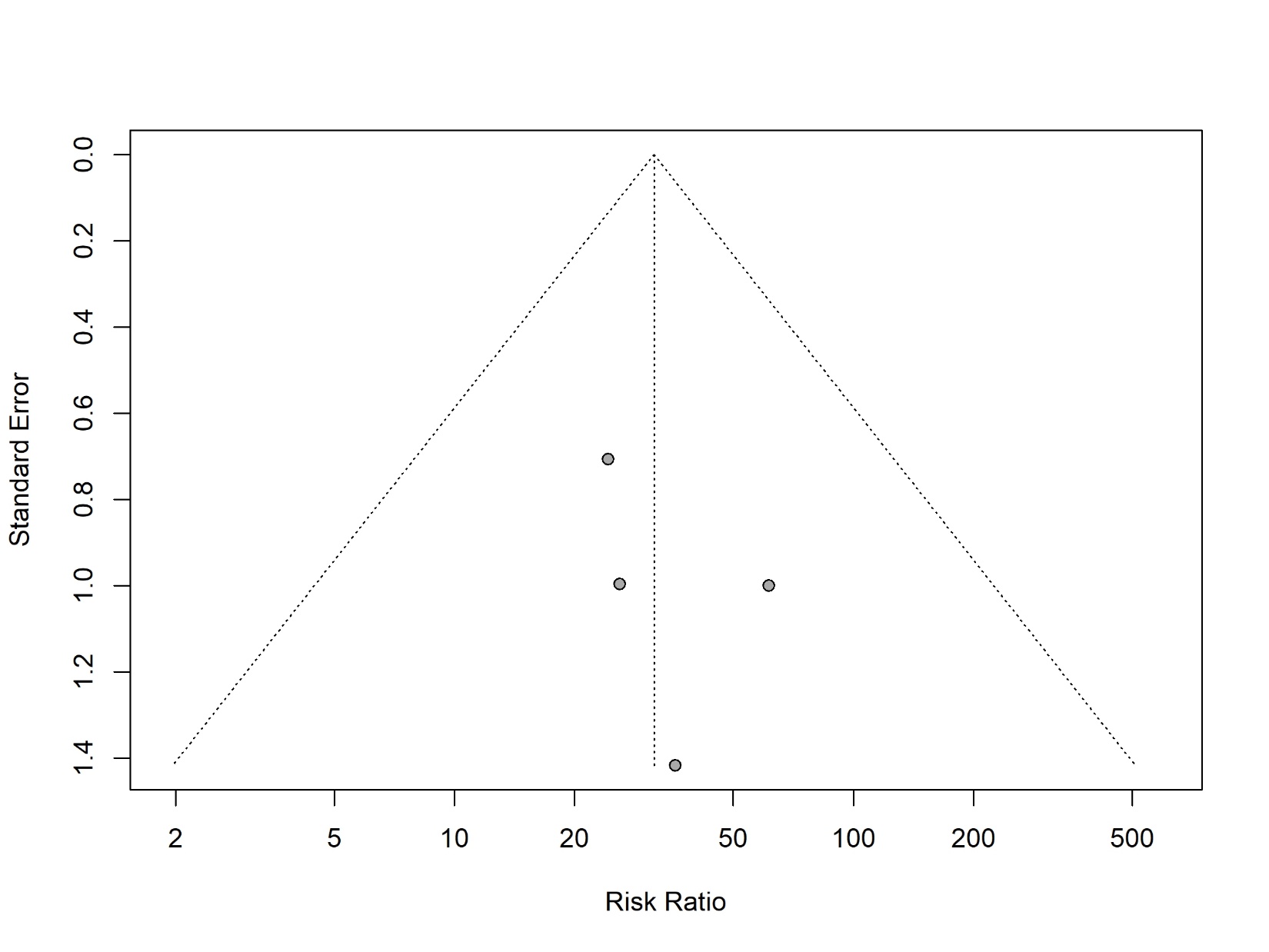


Figure S10: Funnel plot after trim and fill for PASI 100 response at week 16


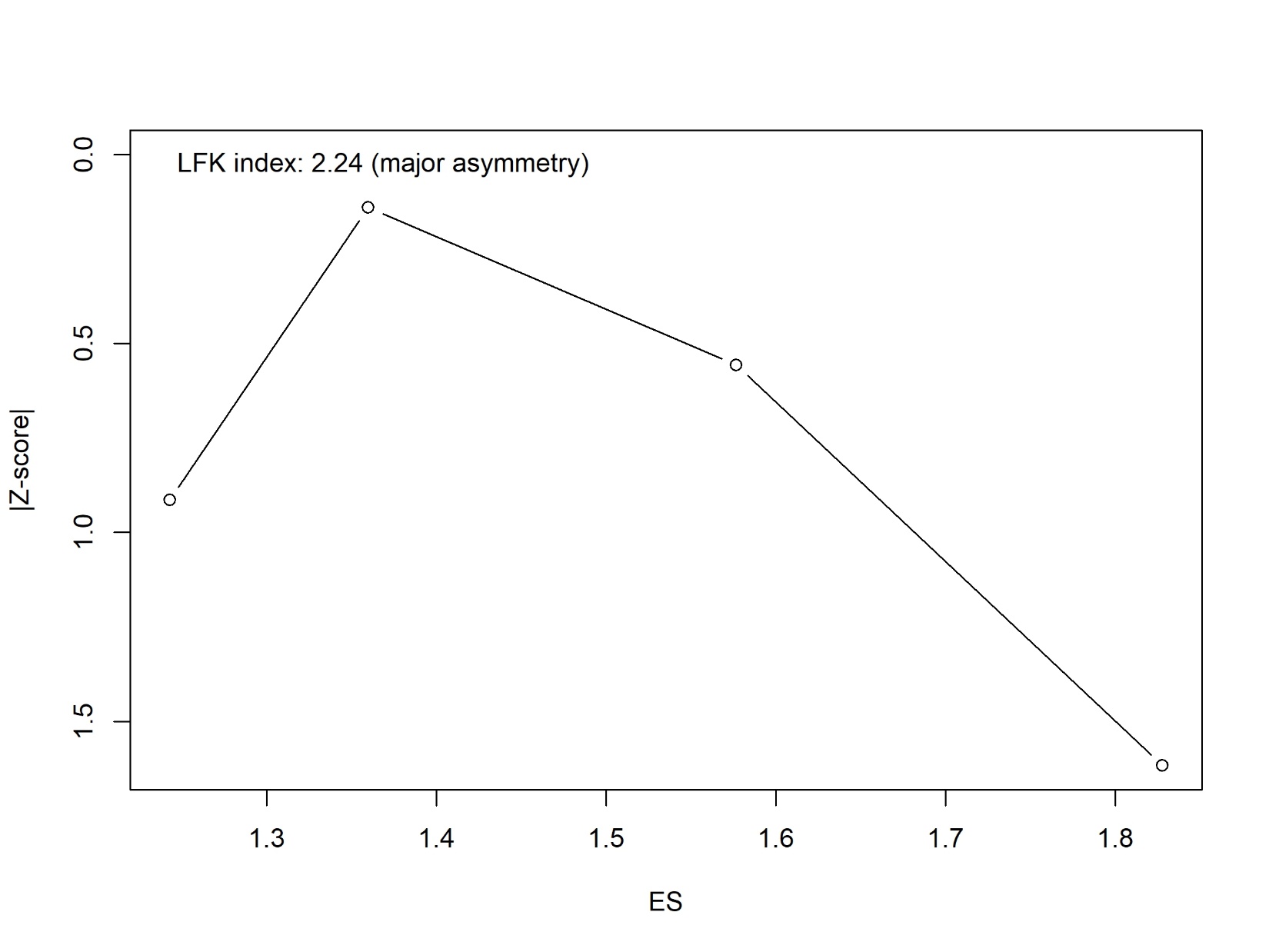


Figure S11: Doi plot for Area Specific Improvement (ss-IGA)


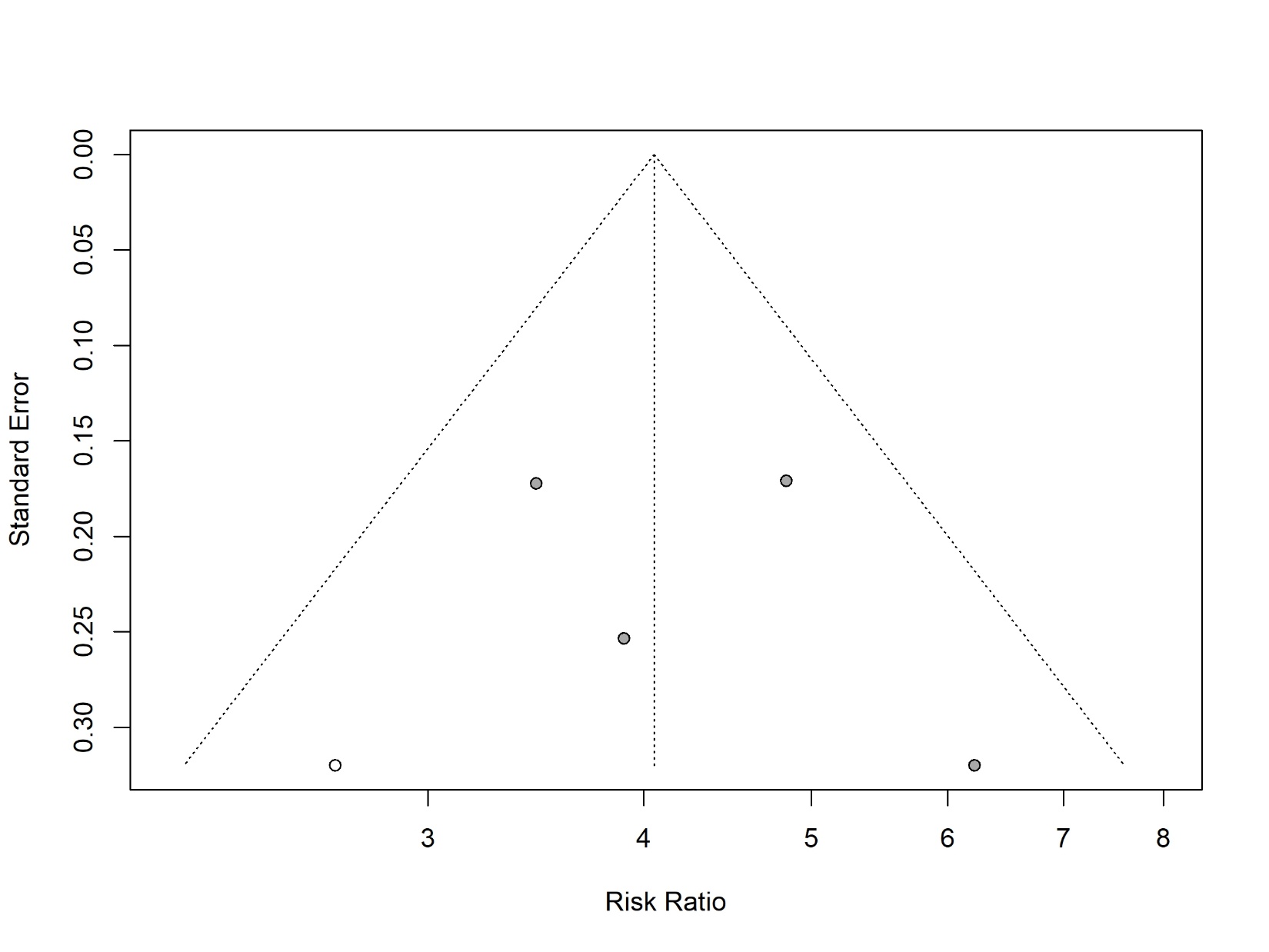


Figure S12: Funnel plot after trim and fill for Area Specific Improvement (ss-IGA)


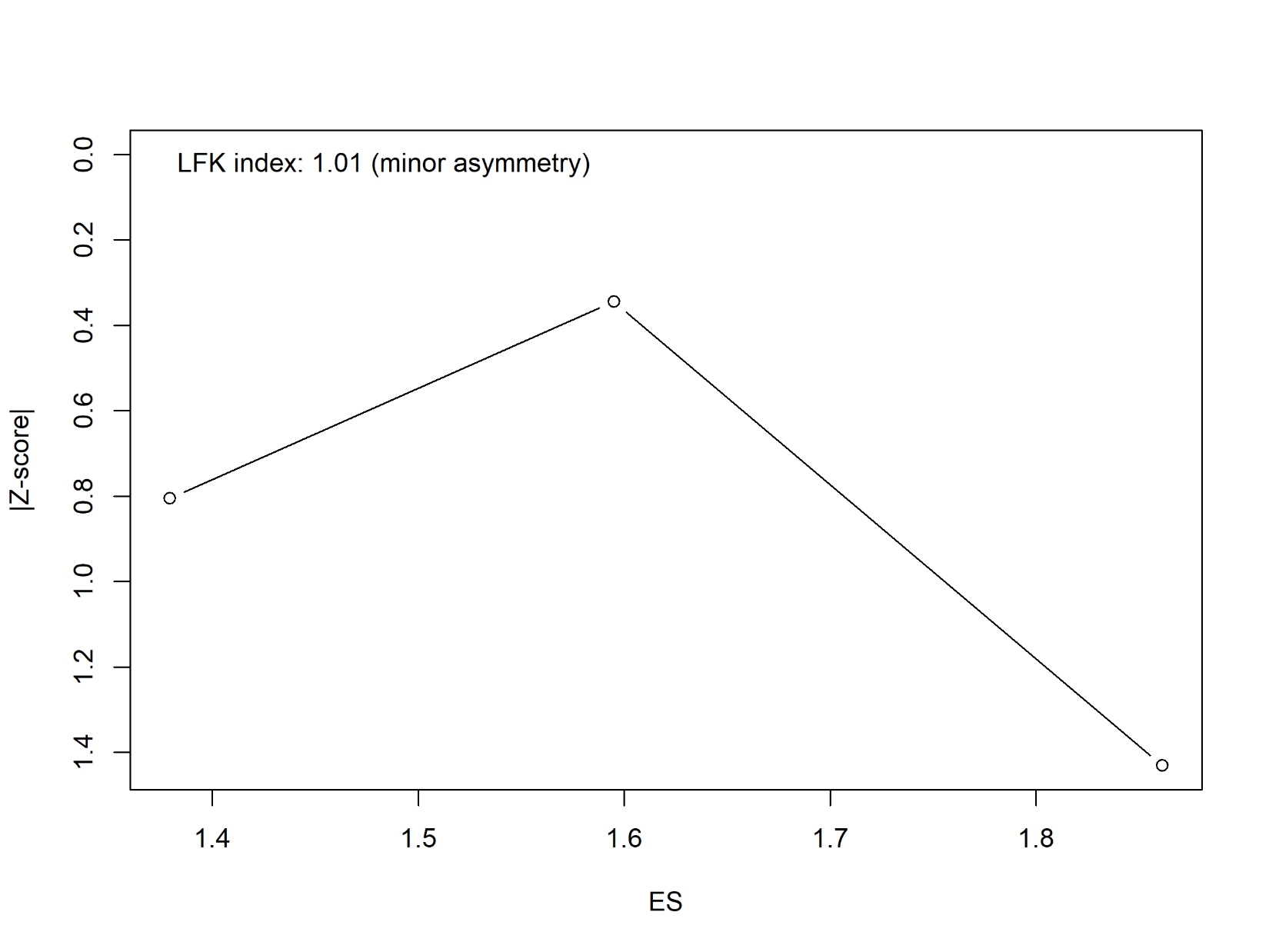


Figure S13: Doi plot for PSSD symptom score of 0 at week 8


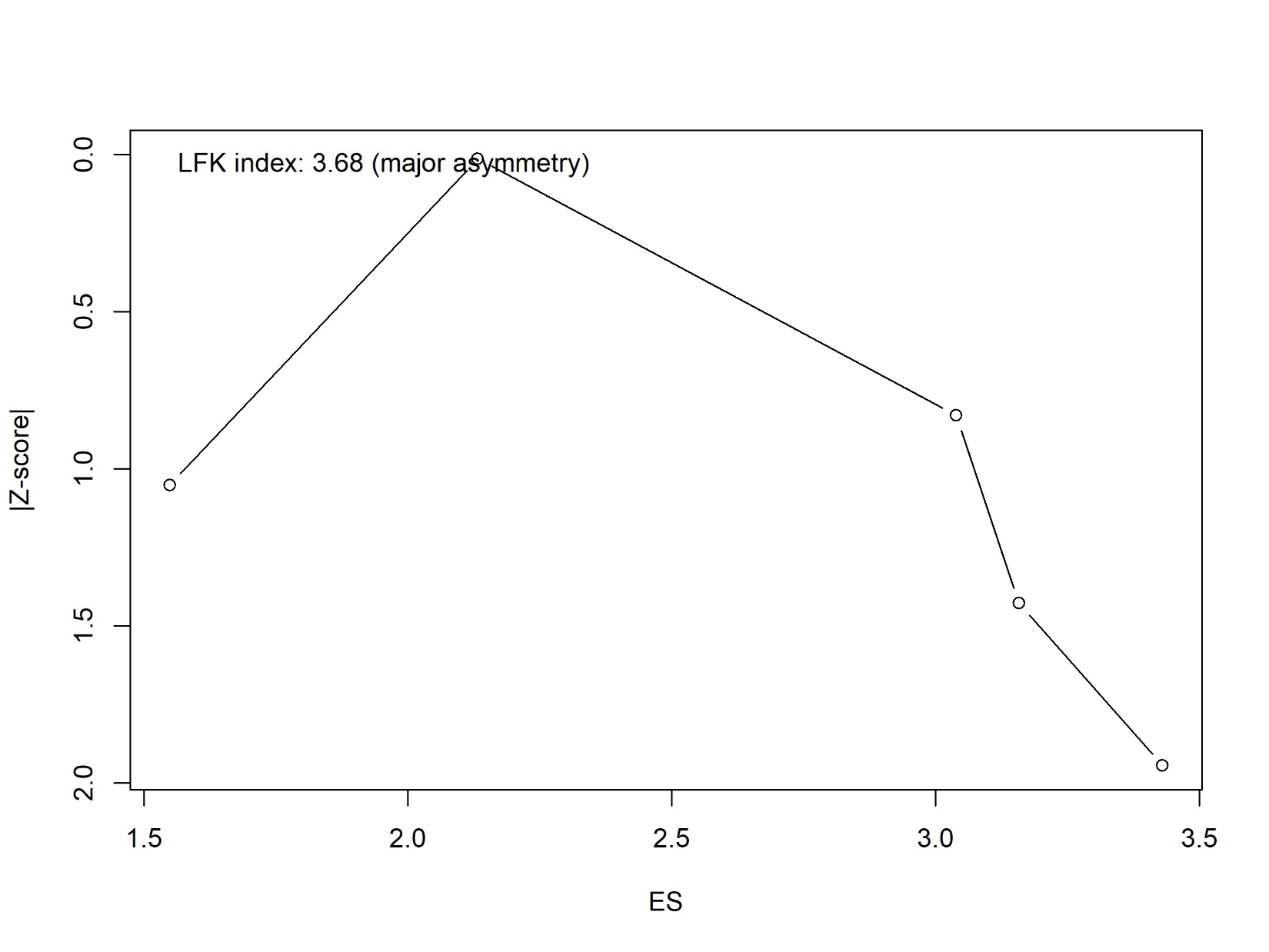


Figure S14: Doi plot for PSSD symptom score of 0 at week 16


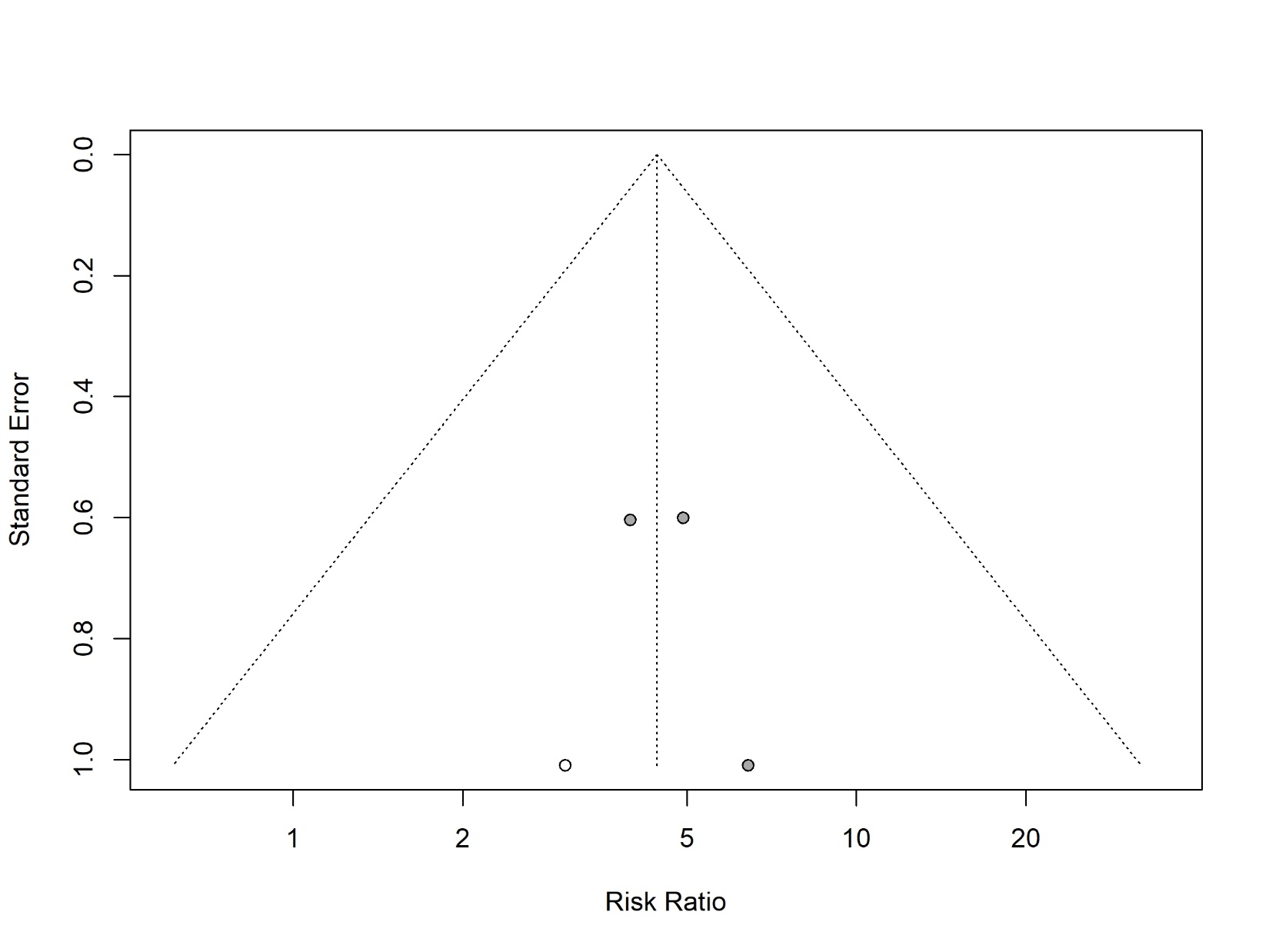


Figure S15: Funnel plot after trim and fill for PSSD symptom score of 0 at week 8


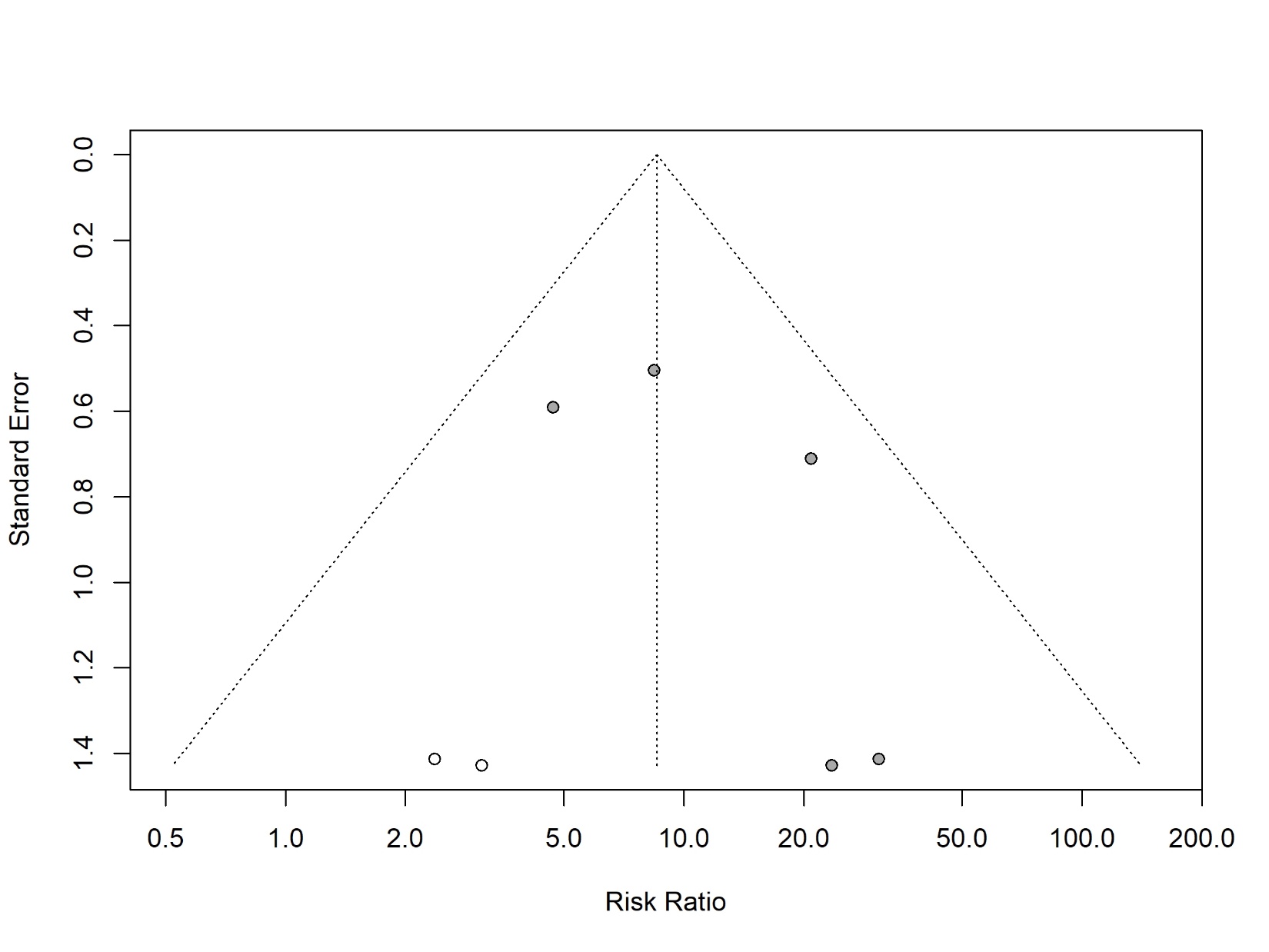


Figure S16: Funnel plot after trim and fill for PSSD symptom score of 0 at week 16


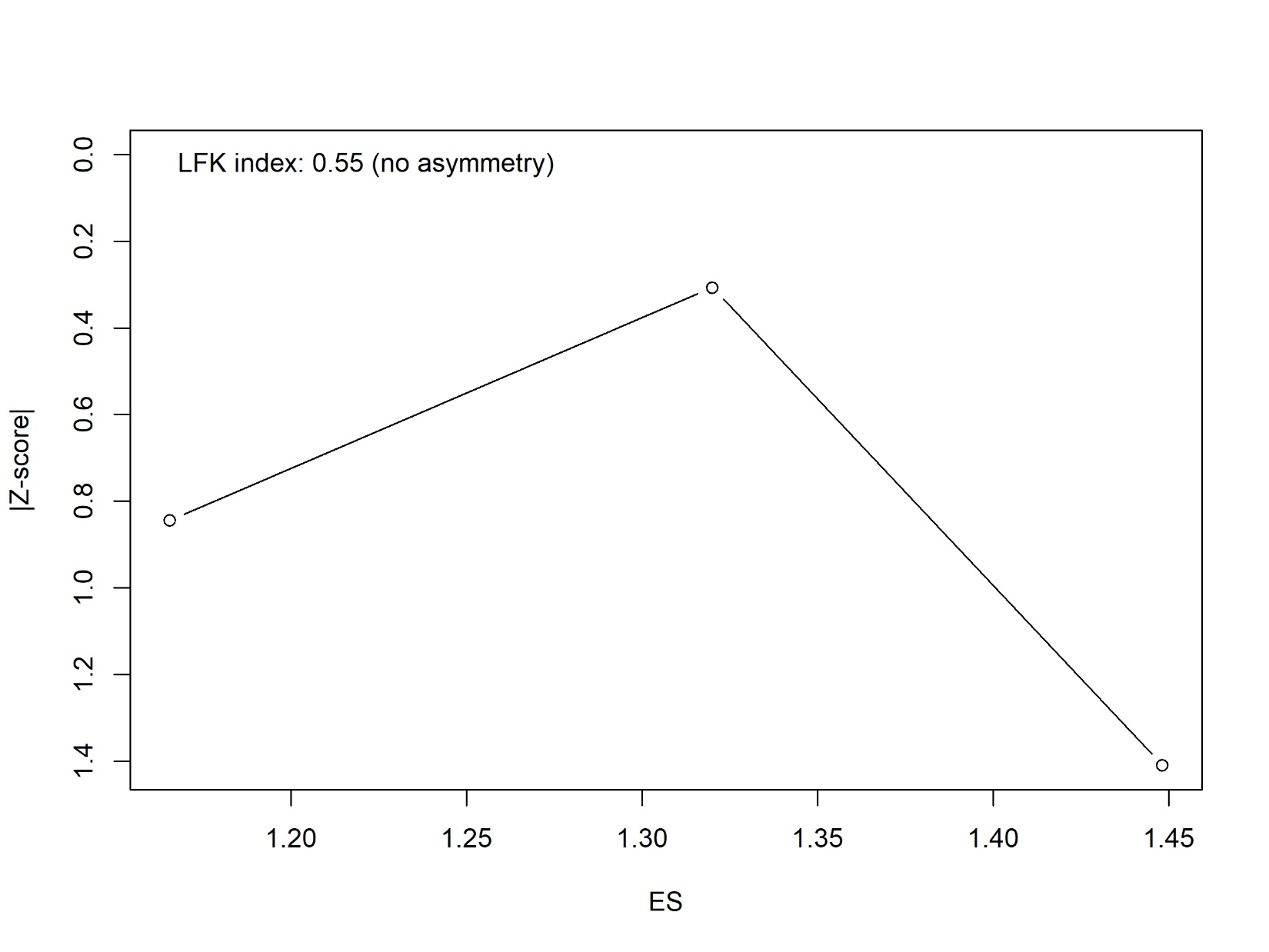


Figure S17: Doi plot for PSSD itch score at week 4


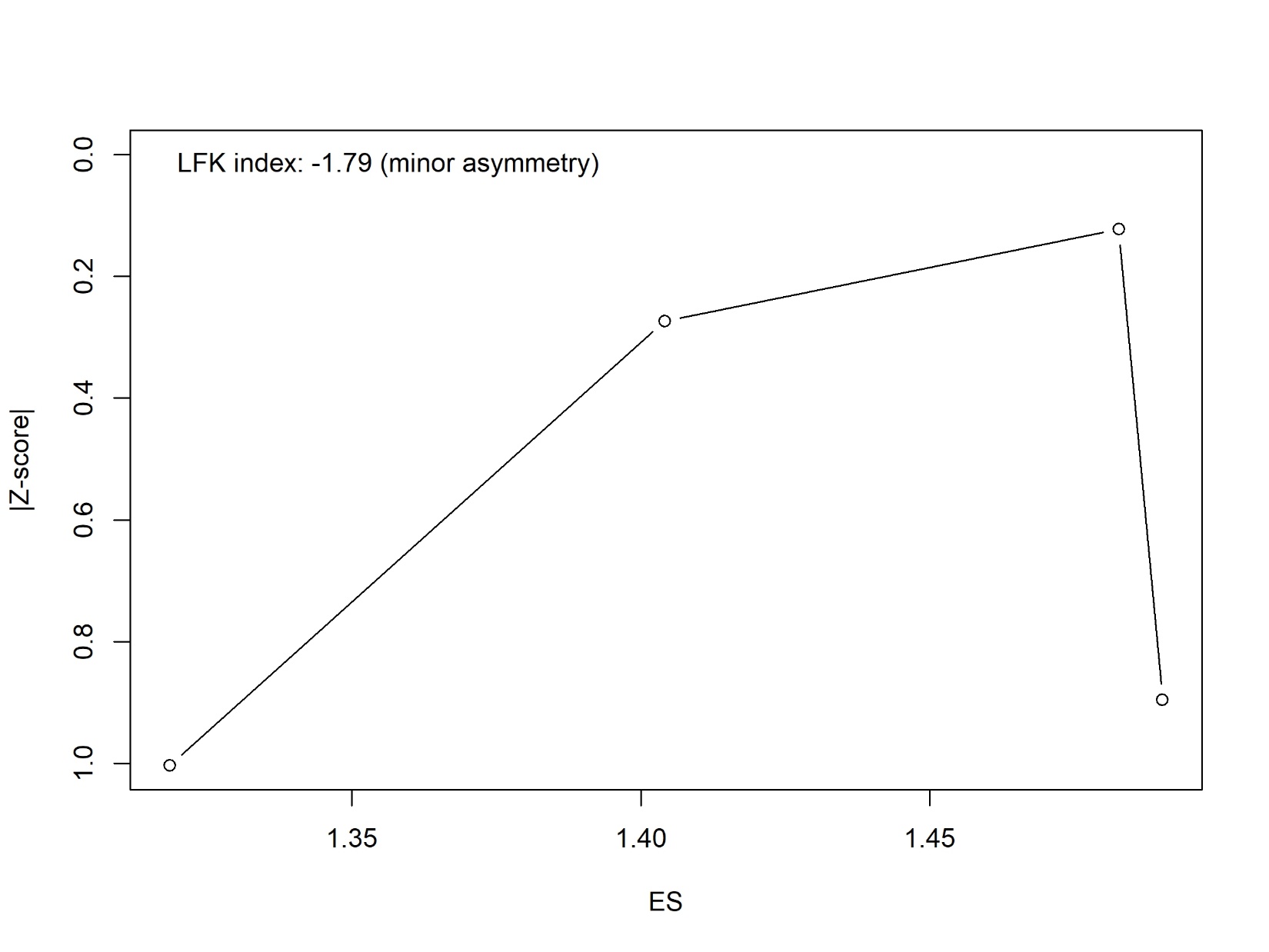


Figure S18: Doi plot for PSSD itch score at week 16


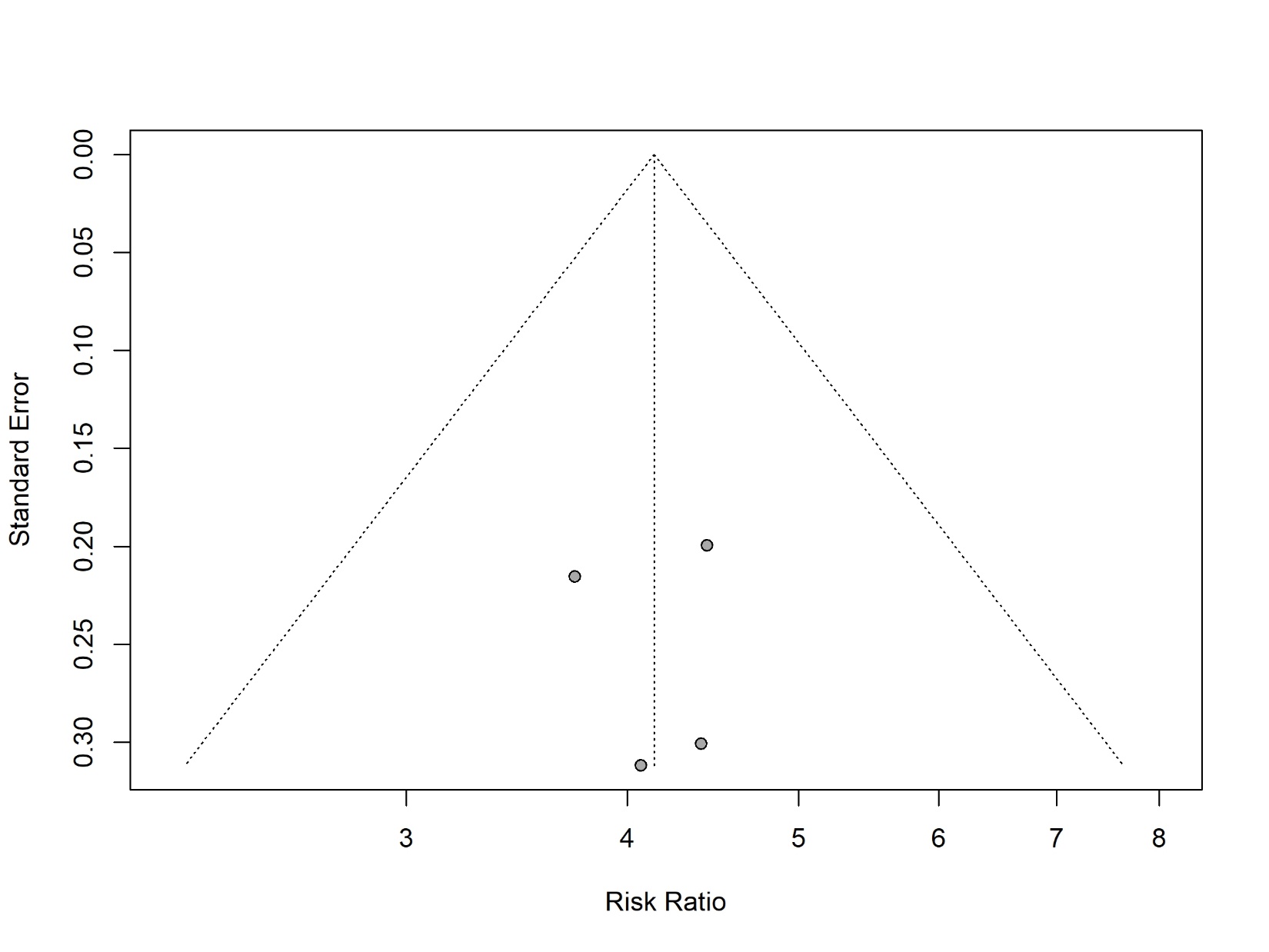


Figure S19: Funnel plot after trim and fill for PSSD itch score at week 16


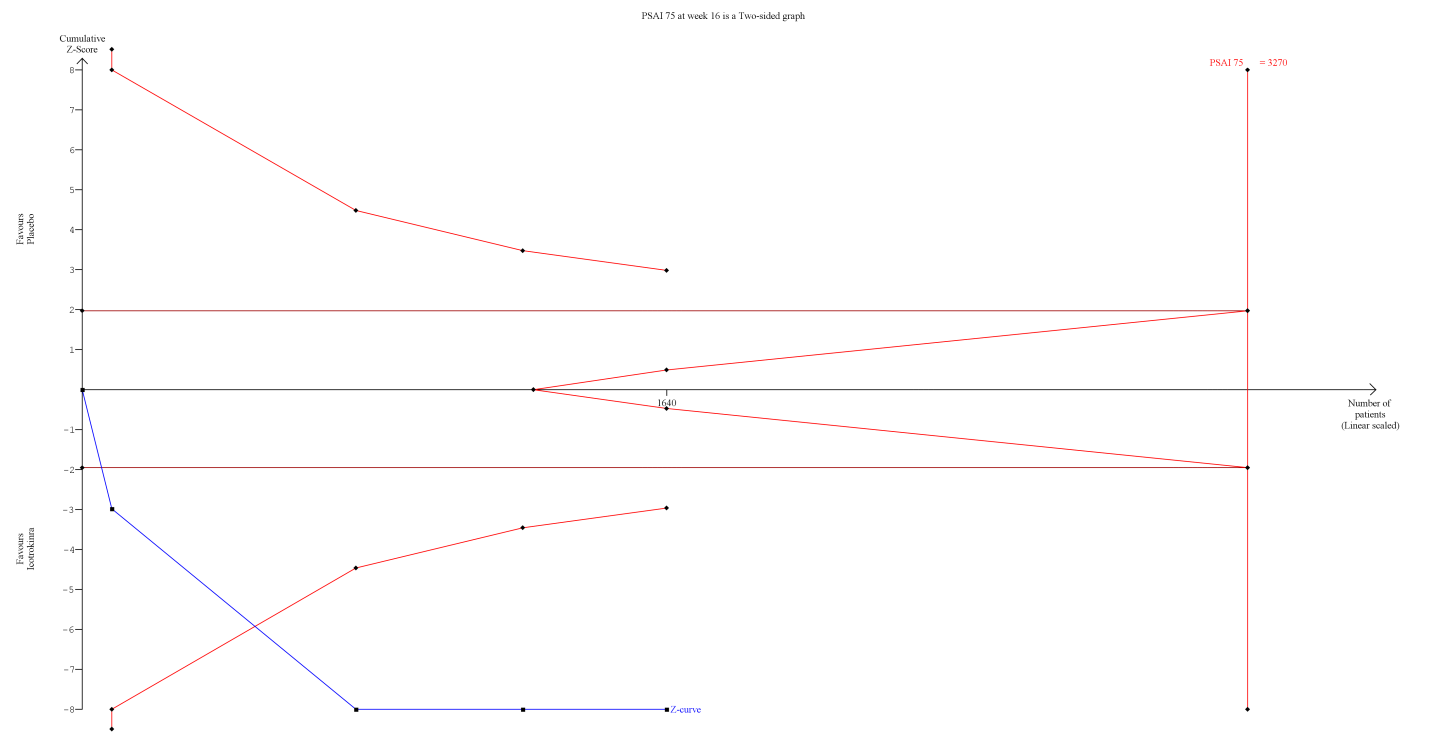


Fig. S20. Trial Sequential Analysis (TSA) for PASI 75 response at week 16.


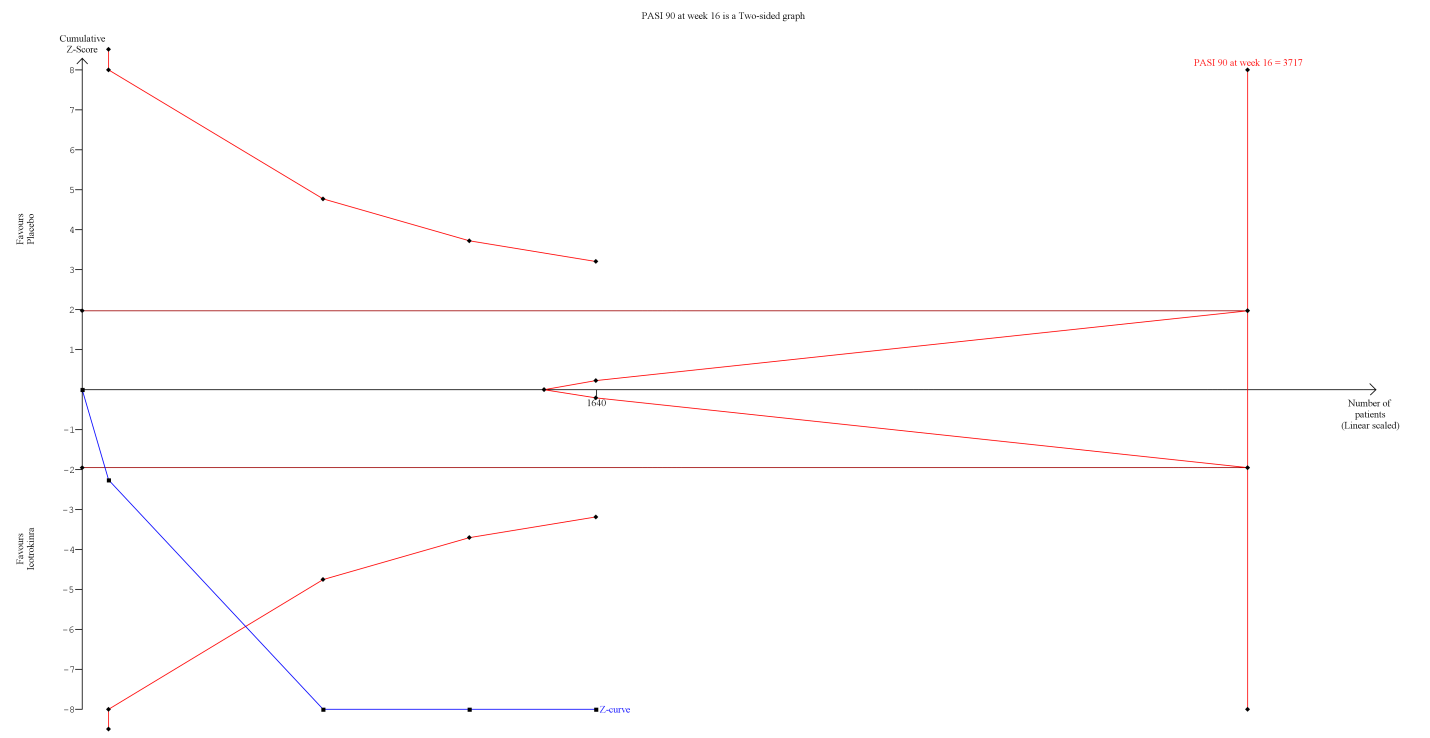


Fig. S21. Trial Sequential Analysis (TSA) for PASI 90 response at week 16.


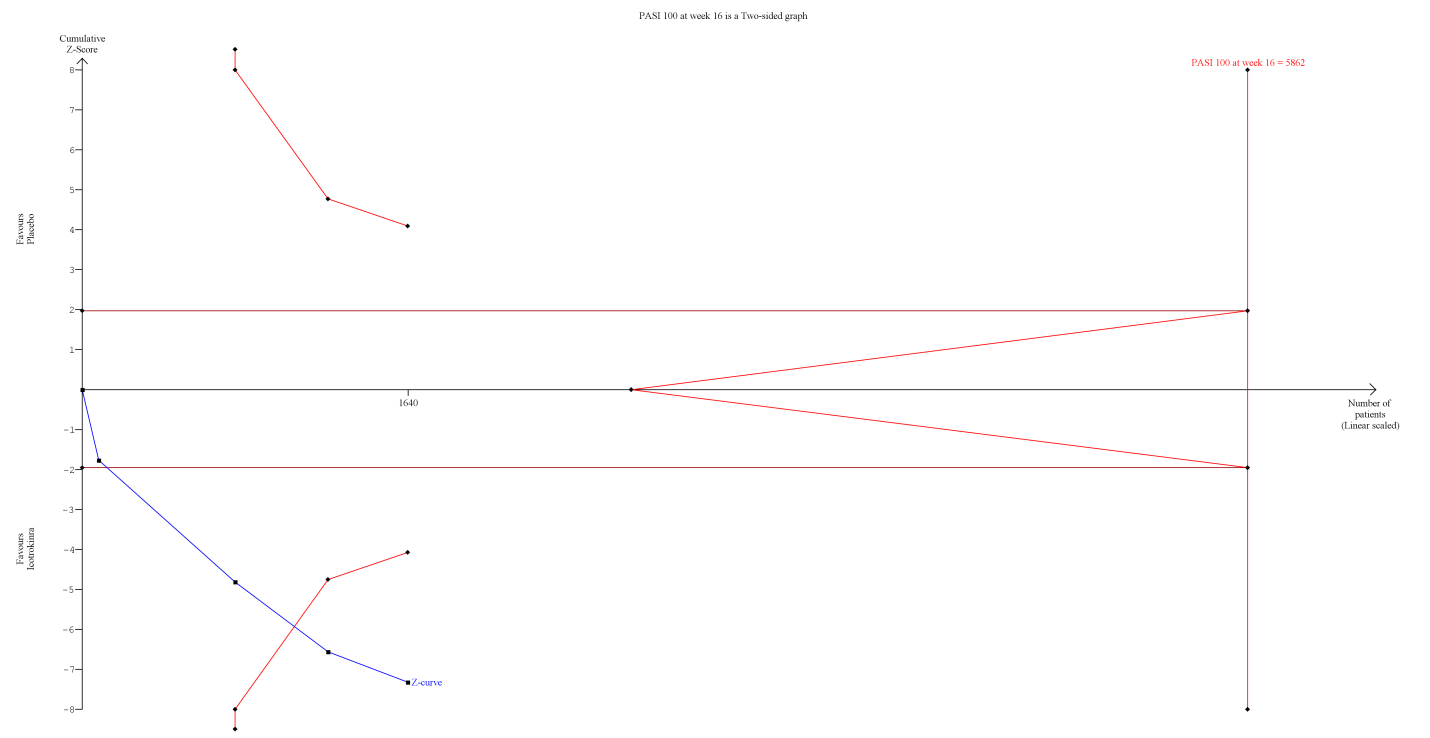


Fig. S22. Trial Sequential Analysis (TSA) for PASI 100 response at week 16.


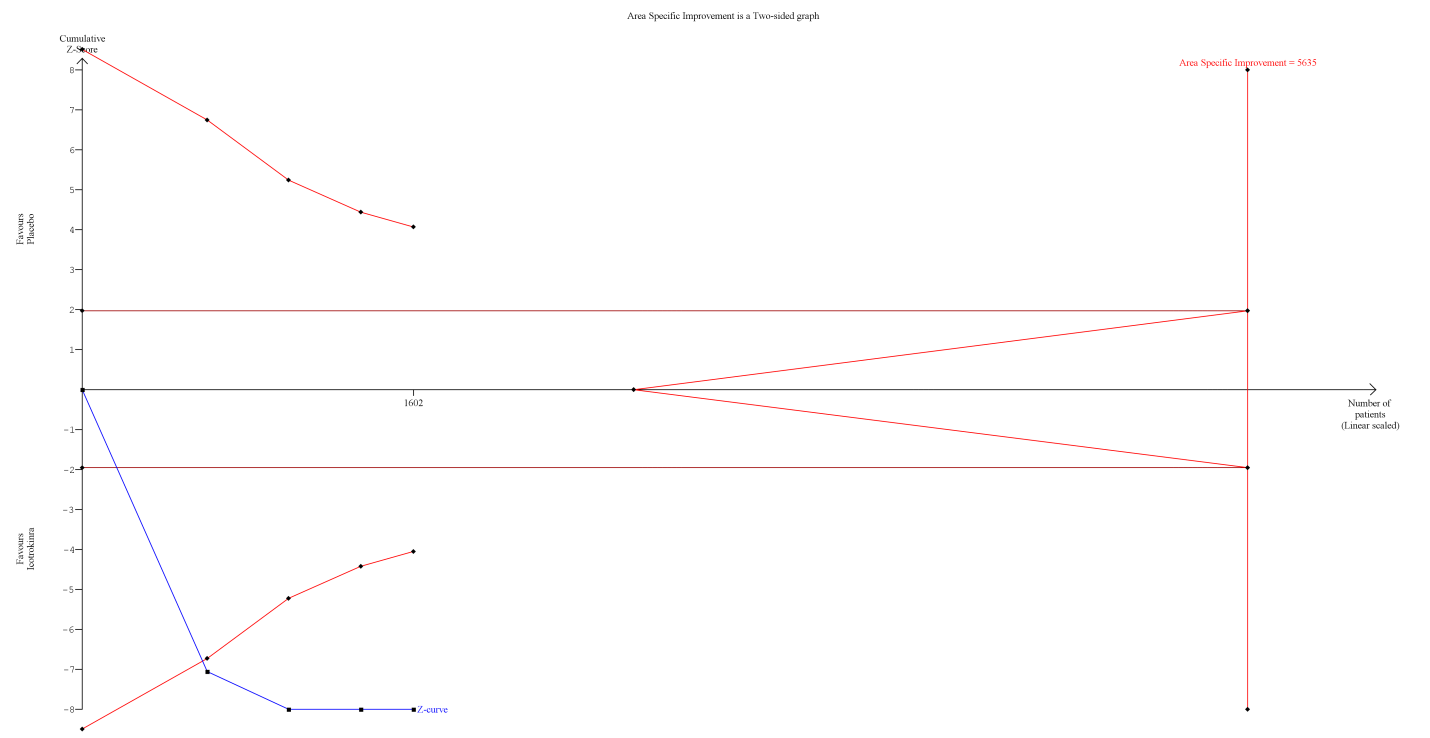


Fig. S23. Trial Sequential Analysis (TSA) for area-specific improvement at week 16.


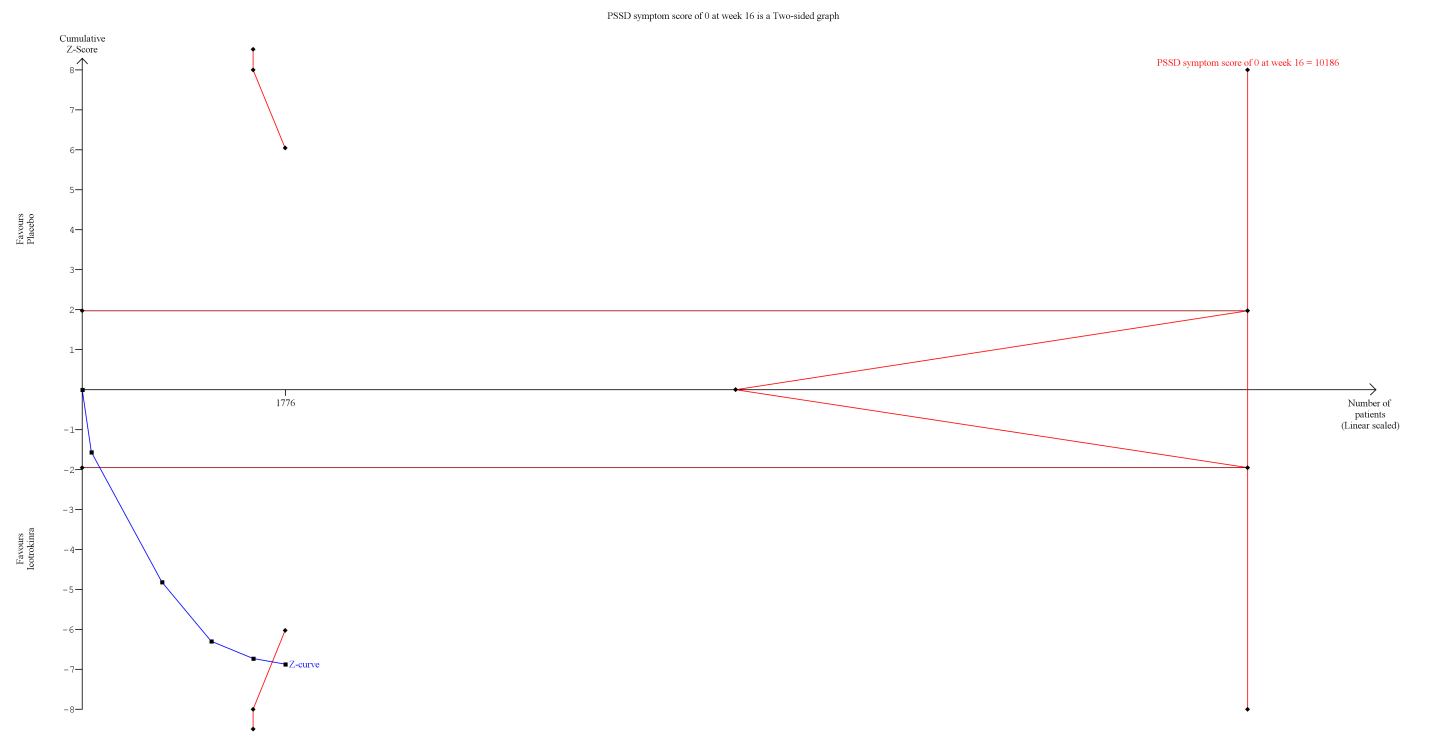


Fig. S24. Trial Sequential Analysis (TSA) for Scalp-specific Investigator's Global Assessment (ss-IGA) 0/1 response at week 16.


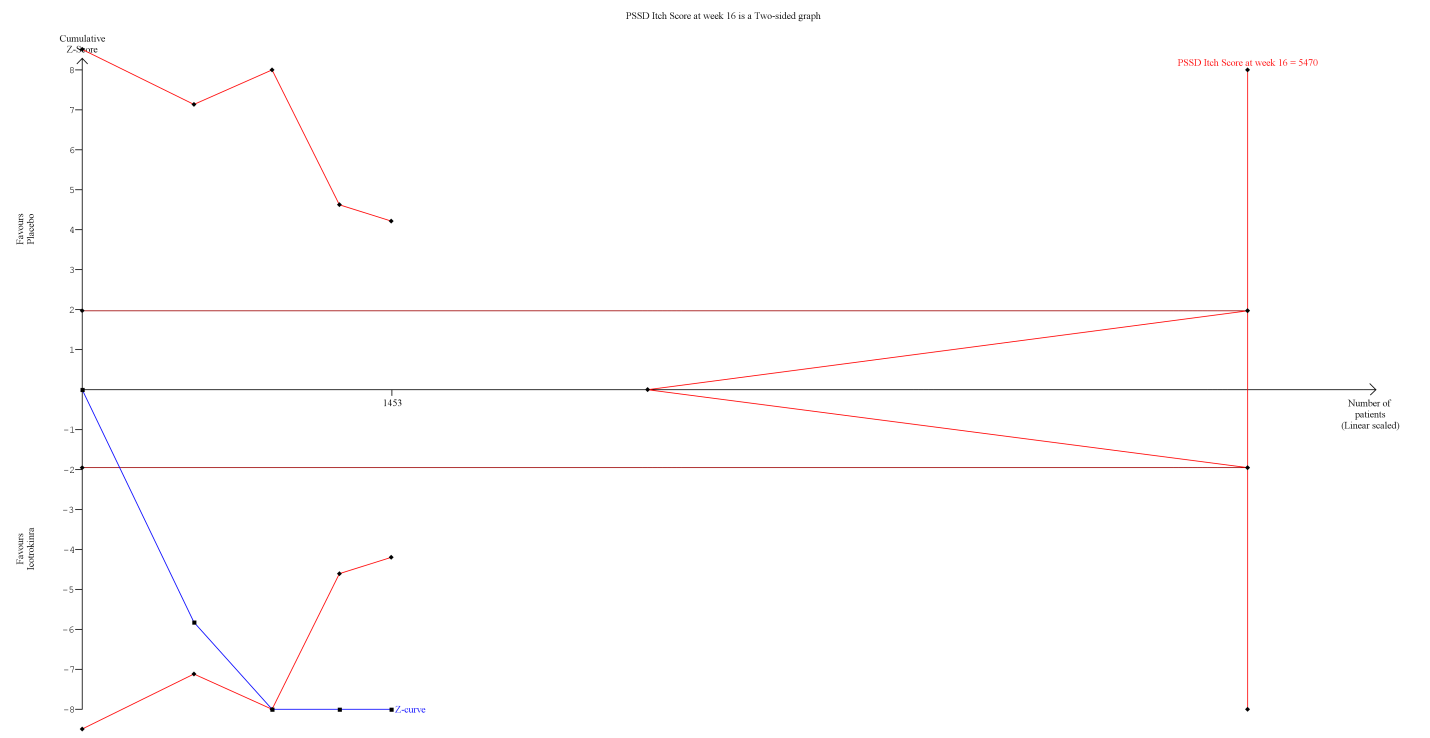


Fig. S25. Trial Sequential Analysis (TSA) for a clinically meaningful improvement in Psoriasis Symptom Scale Diary (PSSD) itch score at week 16.

**PRISMA checklist**

| **Section/topic** | **#** | **Checklist item** | **Reported on page #** |
| --- | --- | --- | --- |
| **TITLE** | | |  |
| Title | 1 | Identify the report as a systematic review, meta-analysis, or both. | 1 |
| **ABSTRACT** | | |  |
| Structured summary | 2 | Provide a structured summary including, as applicable: background; objectives; data sources; study eligibility criteria, participants, and interventions; study appraisal and synthesis methods; results; limitations; conclusions and implications of key findings; systematic review registration number. | 3 |
| **INTRODUCTION** | | |  |
| Rationale | 3 | Describe the rationale for the review in the context of what is already known. | 4-5 |
| Objectives | 4 | Provide an explicit statement of questions being addressed with reference to participants, interventions, comparisons, outcomes, and study design (PICOS). | 5 |
| **METHODS** | | |  |
| Protocol and registration | 5 | Indicate if a review protocol exists, if and where it can be accessed (e.g., Web address), and, if available, provide registration information including registration number. | 5 |
| Eligibility criteria | 6 | Specify study characteristics (e.g., PICOS, length of follow-up) and report characteristics (e.g., years considered, language, publication status) used as criteria for eligibility, giving rationale. | 5 |
| Information sources | 7 | Describe all information sources (e.g., databases with dates of coverage, contact with study authors to identify additional studies) in the search and date last searched. | 5 |
| Search | 8 | Present full electronic search strategy for at least one database, including any limits used, such that it could be repeated. | 5 |
| Study selection | 9 | State the process for selecting studies (i.e., screening, eligibility, included in systematic review, and, if applicable, included in the meta-analysis). | 5 |
| Data collection process | 10 | Describe method of data extraction from reports (e.g., piloted forms, independently, in duplicate) and any processes for obtaining and confirming data from investigators. | 5 |
| Data items | 11 | List and define all variables for which data were sought (e.g., PICOS, funding sources) and any assumptions and simplifications made. | 5 |
| Risk of bias in individual studies | 12 | Describe methods used for assessing risk of bias of individual studies (including specification of whether this was done at the study or outcome level), and how this information is to be used in any data synthesis. | 6 |
| Summary measures | 13 | State the principal summary measures (e.g., risk ratio, difference in means). | 6 |
| Synthesis of results | 14 | Describe the methods of handling data and combining results of studies, if done, including measures of consistency (e.g., I^2^) for each meta-analysis. | 6-7 |

| **Section/topic** | **#** | **Checklist item** | **Reported on page #** |
| --- | --- | --- | --- |
| Risk of bias across studies | 15 | Specify any assessment of risk of bias that may affect the cumulative evidence (e.g., publication bias, selective reporting within studies). | 6 |
| Additional analyses | 16 | Describe methods of additional analyses (e.g., sensitivity or subgroup analyses, meta-regression), if done, indicating which were pre-specified. | - |
| **RESULTS** | | |  |
| Study selection | 17 | Give numbers of studies screened, assessed for eligibility, and included in the review, with reasons for exclusions at each stage, ideally with a flow diagram. | 7 |
| Study characteristics | 18 | For each study, present characteristics for which data were extracted (e.g., study size, PICOS, follow-up period) and provide the citations. | 7 |
| Risk of bias within studies | 19 | Present data on risk of bias of each study and, if available, any outcome level assessment (see item 12). | 7 |
| Results of individual studies | 20 | For all outcomes considered (benefits or harms), present, for each study: (a) simple summary data for each intervention group (b) effect estimates and confidence intervals, ideally with a forest plot. | 8-10 |
| Synthesis of results | 21 | Present results of each meta-analysis done, including confidence intervals and measures of consistency. | 8-10 |
| Risk of bias across studies | 22 | Present results of any assessment of risk of bias across studies (see Item 15). | 7 |
| Additional analysis | 23 | Give results of additional analyses, if done (e.g., sensitivity or subgroup analyses, meta-regression [see Item 16]). | - |
| **DISCUSSION** | | |  |
| Summary of evidence | 24 | Summarize the main findings including the strength of evidence for each main outcome; consider their relevance to key groups (e.g., healthcare providers, users, and policy makers). | 10-13 |
| Limitations | 25 | Discuss limitations at study and outcome level (e.g., risk of bias), and at review-level (e.g., incomplete retrieval of identified research, reporting bias). | 13 |
| Conclusions | 26 | Provide a general interpretation of the results in the context of other evidence, and implications for future research. | 13 |
| **FUNDING** | | |  |
| Funding | 27 | Describe sources of funding for the systematic review and other support (e.g., supply of data); role of funders for the systematic review. | 14 |
